# Neural Probabilistic Circuits: Enabling Compositional and Interpretable Predictions Through Logical Reasoning

**DOI:** 10.1007/s10994-026-07118-7

**Published:** 2026-08-29

**Authors:** Weixin Chen, Simon Yu, Huajie Shao, Lui Sha, Han Zhao

**Affiliations:** 1 Department of Computer Science, University of Illinois Urbana-Champaign, Urbana, IL USA; 2 Department of Electrical and Computer Engineering, University of Illinois Urbana-Champaign, Urbana, IL USA; 3 Department of Computer Science, College of William and Mary, Williamsburg, VA USA

**Keywords:** Interpretability, Compositional models, Probabilistic circuits, Logical reasoning

## Abstract

End-to-end deep neural networks have achieved remarkable success across various domains but are often criticized for their lack of interpretability. While post hoc explanation methods attempt to address this issue, they often fail to accurately represent these black-box models, resulting in misleading or incomplete explanations. To overcome these challenges, we propose an inherently transparent model architecture called Neural Probabilistic Circuits (NPCs), which enable compositional and interpretable predictions through logical reasoning. In particular, an NPC consists of two modules: an attribute recognition model, which predicts probabilities for various attributes, and a task predictor built on a probabilistic circuit, which enables logical reasoning over recognized attributes to make class predictions. To train NPCs, we introduce a three-stage training algorithm comprising attribute recognition, circuit construction, and joint optimization. Moreover, we theoretically demonstrate that an NPC’s error is upper-bounded by a linear combination of the errors from its modules. To further demonstrate the interpretability of NPC, we provide both the most probable explanations and the counterfactual explanations. Empirical results on four benchmark datasets show that NPCs strike a balance between interpretability and performance, achieving results competitive even with those of end-to-end black-box models while providing enhanced interpretability.

## Introduction

End-to-end deep neural networks (DNNs) (Devlin et al., 2019; He et al., 2016; Krizhevsky et al., 2012; Vaswani et al., 2017) have demonstrated remarkable success across various domains (Hinton et al., 2012; Long et al., 2015; Sutskever et al., 2014). However, many of them are black-box models containing complex operators, making it hard to interpret and understand how a decision was made. Although many efforts (Lundberg & Lee, 2017; Ribeiro et al., 2016; Selvaraju et al., 2017) have been made to explain a model’s decision in a post hoc manner, Alvarez-Melis and Jaakkola (2018), Laugel et al. (2019), Slack et al. (2020), and Rudin (2019) show that these explanations are oftentimes not reliable as the explanation model might loosely approximate the underlying model. For example, the explanation model exhibits similar performance to the black-box model but yet relies on entirely different features. Such discrepancy between the explanation model and the black-box model could lead to misleading explanations, e.g., attributing the decision to irrelevant features or missing out important features. Misleading explanations are particularly concerning in high-stake applications such as medical analysis (Hou et al., 2024; Liu et al., 2023) and legal justice (Deeks, 2019; Richmond et al., 2024). Rather than introducing post hoc explanations to explain a black-box model, Rudin (2019) argues that one should create an interpretable model in the first place where each component is designed with a distinct purpose, facilitating an interpretable prediction.

Concept bottleneck models (CBMs) (Koh et al., 2020) aim to enhance interpretability by introducing high-level, human-understandable concepts, such as “red color” and “round shape”, as an intermediate bottleneck, which decomposes a model into two modules: a concept recognition model and a task predictor. The neural-network-based concept recognition model maps the input image to probabilities associated with various concepts. Using these probabilities, the task predictor, typically a linear predictor, produces the probabilities for the various classes. Since the final prediction (i.e., the class with the highest probability) can be interpreted in terms of these concepts, the model’s decision-making process becomes more intuitive for humans to understand. To improve performance on downstream tasks, methods like CEM (Zarlenga et al., 2022), ProbCBM (Kim et al., 2023), and others (Kazhdan et al., 2020; Yeh et al., 2020) change the outputs of the concept recognition model from concept probabilities to concept embeddings. While boosting task performance, such approaches significantly compromise interpretability since the dimensions within concept embeddings lack semantic meanings. On the other hand, to further improve interpretability, some approaches (Barbiero et al., 2023; Ciravegna et al., 2023; Rodríguez et al., 2024) propose architectures for the task predictor that incorporate logical rules, allowing task predictions to be explicitly explained through these rules. However, these logical rules are usually learned from data rather than being predefined by humans, limiting our ability to integrate prior domain knowledge into the model. Additionally, there is currently no theoretical guarantee regarding the performance of the overall model, obscuring the relationship between overall performance and that of the concept recognition model or the task predictor.

To address these challenges, we propose a novel model architecture called Neural Probabilistic Circuits (NPCs), which enable compositional and interpretable predictions through logical reasoning. An NPC comprises two modules: an attribute recognition model and a task predictor. Unlike existing approaches that primarily focus on numerous binary concepts (e.g., “red color”, “yellow color”), we introduce a higher-level categorical characteristic called attributes, which describe the types of concepts (e.g., “color”). This approach reduces the need for additional concept selection or pruning to improve model efficiency (Barbiero et al., 2022; Ciravegna et al., 2023; Zarlenga et al., 2023), while also achieving better performance in concept recognition. Given an input image, the neural-network-based attribute recognition model produces probability vectors for various attributes, with each vector representing the likelihood of various values for the corresponding attribute. These probability vectors serve as inputs to the task predictor, which is implemented using a probabilistic circuit. Probabilistic circuits (Choi et al., 2020; Poon & Domingos, 2011; Zhao et al., 2015, 2016), a type of graphical models (Koller & Friedman, 2009), aim to learn the joint distribution over input variables, in our case, attribute variables and the class variable. During learning, probabilistic circuits embed within their structures and parameters either implicit logical rules learned from data or explicit logical rules predefined by humans. The circuits enable tractable probabilistic reasoning tasks such as joint, marginal, and conditional inferences, thereby revealing relations among the attributes and classes. By leveraging these relations, NPCs can reason over outputs from the attribute recognition model to infer the most probable class. Specifically, the prediction score for a given class is the sum of the likelihood of each combination of attribute values weighted by their relevance to the class. As usual, the final prediction corresponds to the class with the highest score.

Given the compositional nature of NPCs, we propose a three-stage training algorithm. Specifically, the whole procedure involves the following stages: (1) Attribute recognition: We begin by training the attribute recognition model within a multi-task learning framework (Caruana, 1997; Ruder, 2017). (2) Circuit construction: Next, we construct the circuit using two distinct approaches: (i) Data-driven approach learns the circuit’s structure and optimizes its parameters based on data, allowing the underlying logical rules to be embedded within the circuit. (ii) Knowledge-injected approach manually designs the circuit’s structure and assigns its parameters to ensure that human-predefined logical rules are explicitly encoded within the circuit. (3) Joint optimization: Finally, the two modules are jointly optimized in an end-to-end manner to further enhance the overall model’s performance on downstream tasks.

To provide theoretical guarantees regarding the performance of the overall model, we demonstrate that, due to its compositional nature and the use of probabilistic circuits, NPCs exhibit a compositional error bound—the error of the overall model is upper-bounded by a linear combination of the errors from the various modules.

In addition, we provide various types of explanations to make it easier for humans to understand NPC’s predictions: (1) Most Probable Explanation (MPE) identifies the combination of attribute values that contributes most significantly to the predicted class. (2) Counterfactual Explanation (CE) answers the question: Would the model have made the correct prediction had the likelihoods of certain attribute values been adjusted?

Empirical results on four image classification datasets demonstrate NPC’s ability to strike an impressive balance between interpretability and performance on downstream tasks. In particular, NPC outperforms three representative concept-based models and delivers results competitive even with those of an end-to-end deep neural network. Additionally, we perform extensive ablation studies to investigate the advantages of integrating attributes over concepts, along with the impacts of attribute selections, predictor design approaches, and joint optimization.

Our main contributions are as follows,

1. We introduce Neural Probabilistic Circuits (NPCs), a novel model architecture that combines a neural-network-based attribute recognition model and a probabilistic-circuit-based task predictor, enabling compositional and interpretable predictions through logical reasoning.
2. We develop a three-stage training algorithm for NPCs, consisting of (1) attribute recognition through multi-task learning, (2) circuit construction via both data-driven and knowledge-injected approaches, and (3) end-to-end joint optimization.
3. To the best of our knowledge, we are the first to provide a theoretical guarantee on the performance of the compositional bottleneck models, which shows that NPC ’s error can be upper-bounded by a linear combination of the errors from its modules.
4. We provide various types of explanations to facilitate human understanding of NPCs’ predictions, including most probable explanations and counterfactual explanations.
5. We empirically show that NPC demonstrates competitive performance in image classification tasks while providing enhanced interpretability.

## Preliminaries

Probabilistic circuits are a class of graphical models that is used to express a joint distribution over a set of random variables $Z_{1:N}$. A probabilistic circuit $f_{S}$ (henceforth simply referred to as a *circuit*) consists of a rooted directed acyclic graph where leaf nodes are univariate indicators of categorical variables^1^ (i.e., ${\mathbb {I}}(Z_{i}=z_{i}),~z_{i}\in {\mathcal {Z}}_{i},~i\in [N]$) and internal nodes consist of sum nodes and product nodes. Each sum node computes a weighted sum of its children, and each product node computes a product of its children. In an unnormalized circuit, the root node outputs the unnormalized joint probability over variables. Any unnormalized circuit can be transformed into an equivalent, normalized circuit via weight updating (Peharz et al., 2015; Zhao et al., 2015). Hence, without loss of generality, we always assume that $f_{S}$ is normalized; thus, $f_{S}(z_{1:N}) = \Pr (Z_{1:N}=z_{1:N})$. Readers are referred to Sánchez-Cauce et al. (2021) for more details on circuits.

In circuits, the *scope* of a node is defined as the set of variables that have indicators among the node’s descendants, which can be computed recursively—if *v* is a leaf node, say, an indicator over $Z_{i}$, then ${\text {scope}}(v) = \{Z_{i}\}$; otherwise, ${\text {scope}}(v)=\cup _{{\tilde{v}} \in {\text {children}}(v)} ~{\text {scope}}({\tilde{v}})$. A circuit is *smooth* iff each sum node has children with identical scope. A circuit is *decomposable* iff each product node has children with disjoint scopes. If a circuit is smooth and decomposable, then any marginal probability can be computed by setting the leaf nodes corresponding to the marginalized variables to 1. Consequently, inferences are efficient in a circuit as any joint, marginal, or conditional inference can be computed by at most two passes in a circuit. For instance, $\Pr (Z_{1}=z_{1} \mid Z_{2:N}=z_{2:N}) = \frac{\Pr (Z_{1:N}=z_{1:N})}{\Pr (Z_{2:N}=z_{2:N})} = \frac{f_{S}(Z_{1:N}=z_{1:N})}{f_{S}(Z_{1}=\emptyset , Z_{2:N}=z_{2:N})}$ where $Z_{1}=\emptyset$ implies ${\mathbb {I}}(Z_{1}={\tilde{z}}_{1})=1, \forall {\tilde{z}}_{1} \in {\mathcal {Z}}_{1}$; thus, computing a conditional probability only requires two forward processes in a circuit, each in linear time w.r.t. its size. In this paper, we focus on smooth and decomposable circuits.

## Neural Probabilistic Circuits

In this section, we introduce Neural Probabilistic Circuits (NPCs). We begin by describing the model architecture and the inference process, illustrating how NPCs enable compositional and interpretable predictions through logical reasoning (Section 3.1). Next, we elaborate on the three-stage training algorithm for training NPCs. In particular, we propose two distinct approaches for building circuits: a data-driven approach and a knowledge-injected approach (Section 3.2). Finally, we provide a theoretical analysis establishing the relationship between the error of the overall model and those of its individual modules (Section 3.3).

### Model Architecture and Inference

**Fig. 1:**
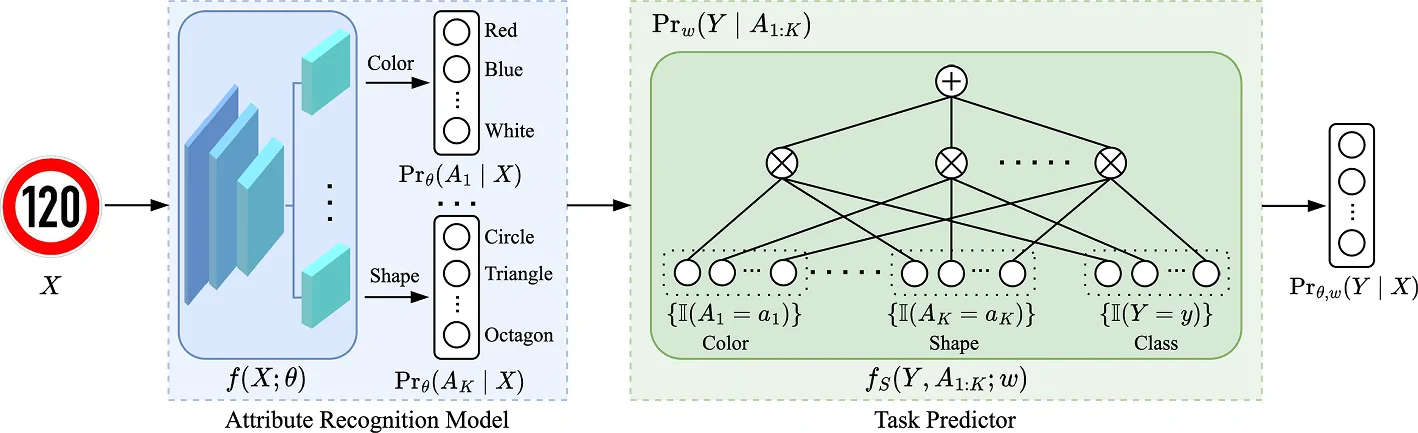
Model architecture of an NPC, consisting of an attribute recognition model and a task predictor. The attribute recognition model is a neural network *f(X;θ )* which takes an image *X* as input and outputs *K* probability vectors $\{\Pr _{\theta }(A_{k}\mid X)\}_{k=1}^{K}$. The task predictor is a probabilistic circuit $f_{S}(Y, A_{1:K}; w)$ taking an instance of attributes as input and providing the conditional probability $\Pr _{w}(Y\mid A_{1:K})$. By leveraging these relations between classes and attributes alongside the probability distributions of various attributes, NPC produces the probability vector $\Pr _{\theta , w}(Y\mid X)$ .

Figure 1 presents an overview of an NPC, which consists of an attribute recognition model and a task predictor. The attribute recognition model is a neural network that processes an input image to identify its high-level visual attributes, such as color and shape. The task predictor is a (normalized) probabilistic circuit that models the joint distribution over attributes and classes, embedding either implicit or explicit logical rules within its structure and parameters during learning. The circuit enables efficient probabilistic reasoning, including joint, marginal, and conditional inferences. Specifically, given a particular assignment of attributes, the circuit can infer the probability of a specific class. By leveraging these conditional dependencies alongside the probability distributions of the various attributes (i.e., outputs from the attribute recognition model), NPC produces the probabilities of the image belonging to various classes. The class with the highest probability is recognized as the predicted class.

Formally, let $X\in {\mathcal {X}}, A_{k}\in {\mathcal {A}}_{k}, Y\in {\mathcal {Y}}$ denote the input variable, the *k*-th attribute variable, and the class variable. The variables’ instantiations are represented by $x, a_{k}, y$, respectively. In particular, we consider *K* attributes, i.e., $A_{1}, \ldots , A_{K}$ (or $A_{1:K}$ in short). Each attribute $A_{k}$ has $q_{k}$ possible values, i.e., $\vert {\mathcal {A}}_{k} \vert = q_{k}$. The attribute recognition model *f(X;θ )* is parameterized by *θ *. Given an input instance *x*, the model outputs *K* probability vectors. The *k*-th probability vector, denoted as $f_{k}(x; \theta ) \in {\mathbb {R}}^{q_{k}}$, shows the probabilities of *x*’s *k*-th attribute taking different values $a_{k}$, i.e., $[f_{k}(x; \theta )]_{a_{k}} = \Pr _{\theta }\left( A_{k}=a_{k} \mid X=x \right)$. The task predictor $f_{S}(Y, A_{1:K}; w)$ is a probabilistic circuit with structure *S* and parameters *w*, which models the joint distribution over $Y, A_{1:K}$. Specifically, when taking an instance of attributes $a_{1:K}$ and a class label *y* as input, the circuit outputs the joint probability $\Pr _{w}(Y=y, A_{1:K} = a_{1:K})$. By the virtue of its efficient inferences, the circuit also supports efficient conditional queries, e.g., $\Pr _{w}\left( Y=y\mid A_{1:K} = a_{1:K} \right) = f_{S}(y, a_{1:K}; w)/f_{S}(\emptyset , a_{1:K}; w)$.

Prior to describing how an NPC predicts a class, we make the following mild assumptions on the selected attributes.

#### Assumption 1

(*Sufficient Attributes*) Given the attributes, the class label is conditionally independent of the input, i.e., $Y\perp X\mid A_{1},\ldots , A_{K}$.

#### Assumption 2

(*Complete Information*) Given any input, all attributes are conditionally mutually independent, i.e., $A_{1}\perp A_{2}\perp \cdots \perp A_{K} \mid X$.

#### Remarks

The first assumption essentially assumes that the attributes are sufficient to infer the class label of interest. The second assumption assumes the input contains complete information regarding the attributes such that they are conditionally mutually independent. These assumptions are mild and often hold in practice. For instance, in the context of traffic signs, if the attributes include the shape (e.g., circle), color (e.g., red), and symbol (e.g., slash) of a sign, they collectively provide enough information to infer the class label (e.g., no entry) without requiring additional details from the raw image. On the other hand, the raw image fully encodes the attributes (e.g., shape, color, and symbol). Once the input is observed, these attributes can be independently determined.

Under Assumption 1 and 2, an NPC outputs the probability of an input *x* being a class *y* as follows,

1 $$\begin{aligned} {\operatorname {Pr}}_{\theta , w}\left( Y=y \mid X=x\right)&=\sum _{a_{1:K}}{\operatorname {Pr}}_{w}\left( Y=y \mid A_{1:K} = a_{1:K}, X=x\right) \cdot {\operatorname {Pr}}_{\theta }\left( A_{1:K} = a_{1:K} \mid X=x\right) \\&=\sum _{a_{1:K}}\underbrace{{\operatorname {Pr}}_{w}\left( Y=y \mid A_{1:K} = a_{1:K}\right) }_{\text {task predictor}} \cdot \underbrace{\prod _{k=1}^{K} {\operatorname {Pr}}_{\theta }\left( A_{k}=a_{k} \mid X=x\right) }_{\text {attribute recognition model}}. \end{aligned}$$

Equation (1) is derived using the assumptions, and the two interior terms are given by the circuit-based task predictor and the attribute recognition model, respectively. Subsequently, the predicted class is the one with the largest probability, i.e., ${\hat{y}} = \arg \max _{y\in {\mathcal {Y}}}~\Pr _{\theta , w}(Y=y \mid X=x)$.

In summary, we propose a novel model architecture for image recognition tasks. The architecture is interpretable by design, thanks to the integration of an attribute bottleneck and the probabilistic semantics of probabilistic circuits. Together, these modules enable predictions which can be interpreted using the likelihood of different attributes and the conditional dependencies between attributes and classes.

### Three-Stage Training Algorithm

In this section, we will propose a three-stage training algorithm for NPCs comprising the following stages: (1) attribute recognition through multi-task learning (Section 3.2.1), (2) circuit construction via both data-driven and knowledge-injected approaches (Section 3.2.2), and (3) joint optimization (Section 3.2.3).

#### Attribute Recognition

We aim to train the attribute recognition model *f(X;θ )* such that each attribute is recognized well. To this end, we adopt a multi-task learning framework (Zhang & Yang, 2021), where each task is to recognize a particular attribute. Specifically, we use the cross-entropy loss for each task and assign weights to the task losses based on the size of the corresponding attribute space. These weights normalize the task losses, preventing certain tasks from dominating the training process (Grégoire et al., 2024; Kendall et al., 2018; Wang & Chen, 2020). The overall training loss for attribute recognition is defined as follows,

2 $$ {\mathcal {L}}_{\text {Attribute}}(\theta ; D) = -\frac{1}{K}\sum _{k}\frac{1}{\log q_{k}} \left( \frac{1}{|D|} \sum _{x\in D}g_{k}^T(x) f_{k}(x;\theta ) \right) . $$

The term inside the parentheses represents the mean cross-entropy loss over the training dataset *D*, where $f_{k}(x;\theta )\in {\mathbb {R}}^{q_{k}}$ and $g_{k}(x)\in {\mathbb {R}}^{q_{k}}$ are, respectively, the output vector and the label vector corresponding to the *k*-th attribute. Specifically, $g_{k}(x)$ is a probability vector that sums to one, with each entry representing the ground-truth likelihood of *x* having a particular attribute value. For instance, for the color attribute, an image of a polar bear would have a one-hot label vector with 1 representing “white”. In contrast, an image of a zebra might have a probabilistic label vector, with 0.5 representing “black” and 0.5 representing “white”.

#### Circuit Construction

We aim to construct a probabilistic circuit $f_{S}(Y, A_{1:K}; w)$ that models the joint distribution over $Y, A_{1:K}$. To achieve this, we propose two distinct approaches for building the circuit’s structure and parameters: a data-driven approach and a knowledge-injected approach.

***Data-Driven Approach***

This approach *learns a circuit’s structure and optimizes its parameters* using data in the form of $\{(y, a_{1:K})\}$. As described above, the training dataset is defined as $D=\{(x, g_{1:K}(x), y)\}$, where each attribute label is represented as a probability vector rather than a single value. For each data, we sample an $a_{k}$ from the categorical distribution defined by $g_{k}(x)$, i.e., $a_{k} \sim {\text {Categorical}}(g_{k}(x)),~k\in [K]$, which results in a processed dataset ${\bar{D}}=\{(x, y, a_{1:K})\}$. **(1) Structure Learning**: LearnSPN (Gens & Domingos, 2013) is a mainstream algorithm for learning a circuit’s structure from data. LearnSPN recursively identifies independent groups to create product nodes, clusters data to form sum nodes, and assigns single variables as leaf nodes. In our approach, we apply LearnSPN on ${\bar{D}}$ to derive a structure tailored to the observed data. **(2) Parameter Learning**: Given the learned structure, optimizing the circuit’s weights (i.e., the weights of edges emanating from sum nodes) is framed as a maximum likelihood estimation (MLE) problem, with the following loss function:

$$ {\mathcal {L}}_{\text {MLE}}(w; D) = -\sum _{(y, a_{1:K})\in {\bar{D}}} \log f_{S}\left( y, a_{1:K}; w \right) . $$

We adopt the widely used CCCP algorithm (Zhao et al., 2016) which iteratively applies multiplicative weight updates on *w* to minimize ${\mathcal {L}}_{\text {MLE}}(w; {\bar{D}})$. CCCP is guaranteed to converge monotonously. Overall, with the learned structure and optimized parameters, the circuit captures the underlying logical rules present in the observed data, thus effectively modeling the joint distribution over attributes and classes.

**Fig. 2:**
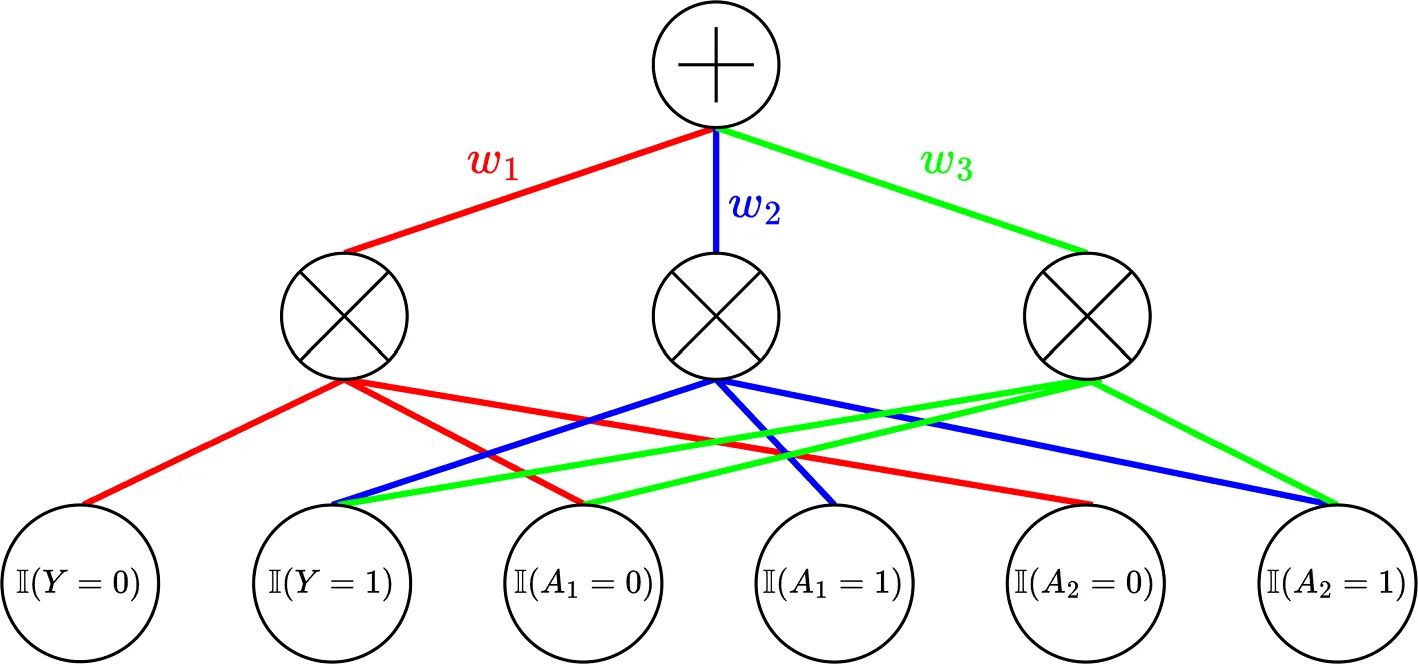
Illustration of a probabilistic circuit constructed by the knowledge-injected approach. The circuit encodes the set of weighted logical rules: $\{w_{1} \cdot \left( {\mathbb {I}}\left( A_{1}=0\right) \wedge {\mathbb {I}}\left( A_{2}=0\right) \wedge {\mathbb {I}}\left( Y=0\right) \right) ,~w_{2} \cdot \left( {\mathbb {I}}(A_{1}=1) \wedge {\mathbb {I}}(A_{2}=1) \wedge {\mathbb {I}}(Y=1) \right) ,~w_3 \cdot \left( {\mathbb {I}}\left( A_{1}=0\right) \wedge {\mathbb {I}}\left( A_{2}=1\right) \wedge {\mathbb {I}}\left( Y=1\right) \right) \}$.

***Knowledge-Injected Approach***

Incorporating domain knowledge into a model helps ensure that its behavior aligns with the human’s understanding of the domain. In practice, domain knowledge can be represented as a set of weighted logical rules. These rules are usually derived by observing patterns in existing samples, with the weight of each rule reflecting the frequency with which the rule holds true among the observed data. For instance, in the context of traffic signs, a rule could be: ${\mathbb {I}}({\text {shape}}={\text {circle}}) \wedge {\mathbb {I}}({\text {color}}={\text {red}}) \wedge {\mathbb {I}}({\text {symbol}}={\text {slash}}) \wedge {\mathbb {I}}({\text {class}}={\text {no entry}})$, as this holds true for an image of a “no entry” sign. The rules summarized for various tasks, along with the process of establishing them, are detailed in Appendix 5. By leveraging these rules, the knowledge-injected approach *manually constructs a circuit’s structure and assigns its parameters* to model the joint distribution over $Y, A_{1:K}$. **(1) Structure Construction**: We construct the structure of the circuit to be a depth-2 weighted sum-of-products formula. More specifically, consider *L* rules of the form $\{r_{l}:= {\mathbb {I}}(A_{1}=a_{1}^{l}) \wedge \ldots \wedge {\mathbb {I}}(A_{K}=a_{K}^{l}) \wedge {\mathbb {I}}(Y=y^{l})\}_{l=1}^{L}$. The structure is constructed as follows: (i) A set of leaf nodes is created to represent the indicator variables of $Y, A_{1:K}$. (ii) Based on these leaf nodes, a layer of product nodes is built, where each product node is associated with a rule. Specifically, the *l*-th product node connects to the leaf nodes that represent the conditions in the rule $r_{l}$, i.e., ${\mathbb {I}}(A_{1}=a_{1}^{l}), \ldots , {\mathbb {I}}(A_{K}=a_{K}^{l}), {\mathbb {I}}(Y=y^{l})$. (iii) A single sum node, which serves as the root node of the circuit, is placed above the product node layer. This sum node aggregates the outputs of all product nodes. **(2) Parameter Assignment**: The parameters in the circuit refer to the weights of edges connecting the product nodes to the sum node. The weight for the *l*-th edge is assigned as the frequency of the rule $r_{l}$. An example of a circuit constructed using this approach is illustrated in Fig. 2. Through these two steps, the human-predefined logical rules are manually encoded into the circuit’s structure and parameters. Proposition 1 ensures that the output of the circuit’s root node represents the empirical joint probability over attributes and classes.

##### Proposition 1

The circuit constructed using the knowledge-injected approach models the empirical joint distribution over attributes and classes. Specifically, the output of the root node represents the empirical joint probability of attributes and classes.

##### Proof

Let *R* denote the rule variable. For a given instance $(y, a_{1:K})$, the output of the root node is computed as,

$$\begin{aligned} f_{S}(y, a_{1:K}; w)&= \sum _{l} \Pr _{w}(R=r_{l})\cdot {\mathbb {I}}(a_{1}=a_{1}^{l})\ldots {\mathbb {I}}(a_{K}=a_{K}^{l})\cdot {\mathbb {I}}(y=y^{l}) \\&= \sum _{l} \Pr _{w}(R=r_{l})\cdot \Pr _{w}(A_{1:K}=a_{1:K}, Y=y \mid R=r_{l}) \\&= \Pr _{w}(A_{1:K}=a_{1:K}, Y=y), \end{aligned}$$

where $\Pr _{w}(R=r_{l})$*l* is the weight for the -th edge and ${\mathbb {I}}(a_{1}=a_{1}^{l})\ldots {\mathbb {I}}(a_{K}=a_{K}^{l})\cdot {\mathbb {I}}(y=y^{l})$ is the output of the *l*-th product node. The condition $R=r_{l}$ indicates that the rule ${\mathbb {I}}(A_{1}=a_{1}^{l}) \wedge \cdots \wedge {\mathbb {I}}(A_{K}=a_{K}^{l}) \wedge {\mathbb {I}}(Y=y^{l})$ is satisfied. *□ *

#### Joint Optimization

Thanks to the differentiability of circuits, NPCs can be fine-tuned in an end-to-end manner to further improve the performance of the overall model on downstream tasks. Specifically, the loss function is defined as follows,

3 $$ {\mathcal {L}}_{\text {Joint}}(\theta , w; (x, y)) = -\sum _{(x, y)\in D}\log {\operatorname {Pr}}_{\theta , w} (Y=y \mid X=x). $$

To optimize this loss, we simply employ the stochastic gradient descent algorithm for updating *θ *, while the projected gradient descent algorithm is used to update *w* to ensure the positivity of the circuit weights. The detailed optimization process is provided in Appendix 1.

### Theoretical Analysis

In this section, we present an error analysis for NPCs to understand how the performance of individual modules affects that of the overall model. Given the overall model and the attribute recognition model are discriminative models, while the probabilistic circuit is a generative model, we define the following errors: **(1) Error of the overall model**: $\epsilon _{\theta , w}:= {\mathbb {E}}_{X}\left[ d_{\text {TV}}(\Pr _{\theta , w}(Y\mid X), \Pr (Y\mid X)) \right]$, which represents the expected total variance distance between the learned and true conditional distributions of *Y* given *X*. **(2) Error of the attribute recognition model**: $\epsilon _{\theta }:= {\mathbb {E}}_{X}\left[ d_{\text {TV}}(\Pr _{\theta }(A_{1:K}\mid X), \Pr (A_{1:K}\mid X)) \right]$, which quantifies the expected total variation distance between the learned and true conditional distributions of the attributes $A_{1:K}$ given *X*. Additionally, we define $\epsilon _{\theta }^{k}:= {\mathbb {E}}_{X}\left[ d_{\text {TV}}(\Pr _{\theta }(A_{k}\mid X), \Pr (A_{k}\mid X)) \right]$ as the error for each individual attribute $A_{k}$. **(3) Error of the probabilistic circuit**: $\epsilon _{w}:= d_{\text {TV}}(\Pr _{w}(Y, A_{1:K}), \Pr (Y, A_{1:K}))$, which measures the total variation distance between the learned and true joint distributions of *Y* and $A_{1:K}$. The above errors capture how closely the learned models approximate the underlying true distributions.

#### Theorem 2

(Compositional Error) Under Assumptions 1 and 2, the error of an NPC is bounded by a linear combination of the errors of the attribute recognition model and the circuit-based task predictor. In particular, the error of the attribute recognition model across all attributes is bounded by the sum of the errors for each attribute, i.e.,

$$ \epsilon _{\theta , w} \leqslant \epsilon _{\theta } + 2\epsilon _{w} \leqslant \sum _{k=1}^{K} \epsilon _{\theta }^{k} + 2\epsilon _{w}. $$

#### Proof Sketch

By leveraging Eq. (1), the upper bound of $\epsilon _{\theta , w}$ is decomposed into two terms. The first term depends only on *θ * and represents the error of the attribute recognition model, while the second term depends only on *w* and captures the error of the circuit. In particular, the overall error of the attribute recognition model is further expanded into the errors across individual attributes. *□ *

The complete proof is deferred to Appendix 2. Theorem 2 demonstrates that reducing the error for any single attribute helps reduce the overall error of the attribute recognition model. More importantly, the error bound of an NPC is decomposable into contributions from its individual modules, which accredits to the compositional nature of NPCs and the incorporation of probabilistic circuits. Consequently, reducing the error of any individual module helps improve the performance of the NPC.

## Model Explanations

As discussed in Section 3.1, model predictions can be interpreted using the attribute recognition results and the conditional dependencies between classes and attributes. To further enhance the human’s understanding of the model predictions, we provide various explanations addressing the following questions: *(1) Which assignment of attributes contributes most to the model prediction?* *(2) In cases where the model prediction was incorrect, could an adjustment to the attribute recognition results lead to a correct prediction?* With a slight abuse of notation, let *θ * denote the parameters of the trained attribute recognition model and let *S*, *w* denote the structure and parameters of the constructed probabilistic circuit.

### Most Probable Explanations

To address the first question, we define Most Probable Explanations (MPEs) for NPCs for identifying the highest contributing attribute assignments.

#### Definition 1

(*Most Probable Explanations*) Given an input *x* with prediction ${\hat{y}}$, the most probable explanation is defined as the attribute assignment that contributes the most in $\Pr _{\theta , w}(Y={\hat{y}} \mid X=x)$. Formally, $a_{1:K}^{*}:= \arg \max _{a_{1:K}}{\operatorname {Pr}}_{w}\left( Y={\hat{y}} \mid A_{1:K} = a_{1:K}\right) \cdot \prod _{k=1}^{K} {\operatorname {Pr}}_{\theta }\left( A_{k}=a_{k} \mid X=x\right)$.

MPE inference is generally challenging for probabilistic circuits. Such inference is tractable for selective circuits (Sánchez-Cauce et al., 2021), but this type of circuit is relatively restrictive in expressiveness. As the number of attributes is small in our experimental settings, we simply use the brute-force algorithm to infer MPEs. Developing more efficient heuristics for MPE inference remains an open problem and is not the primary focus of this paper. Therefore, we leave it for future work.

MPEs provide a concrete explanation as to how the model arrives at a specific class prediction. Specifically, the predicted class is primarily due to the input image’s attributes being recognized as $a_{1:K}^{*}$. These explanations offer attribute-level insights into the model’s predictions, thereby enhancing interpretability and the human’s understanding of the predictions.

To gain deeper insights into how these explanations represent the model’s behavior, we define a property for MPEs, called *alignment*, and introduce a corresponding metric to characterize the behavior of the model.

#### Definition 2

(*Alignment Rate*) An MPE $a_{1:K}^{*}$ is considered aligned with a sample *x* if $a_{1:K}^{*}$ matches the ground-truth attribute assignments of *x*, i.e., $a_{k}^{*} \in \{ j\in [q_{k}] \mid g_{k}^j(x)>0 \},~k\in [K]$. The alignment rate is defined as the proportion of aligned MPEs among all correctly predicted samples.

A high alignment rate reflects strong model reliability, as it suggests that the ground-truth attribute assignments contribute the most during prediction. In other words, the model closely adheres to the human’s understanding when making predictions.

### Counterfactual Explanations

To address the second question, we define Counterfactual Explanations (CEs) (Wachter et al., 2017) for NPCs to explore admissible changes in attribute recognition results that can correct any incorrectly predicted classes.

#### Definition 3

(*Counterfactual Explanations*) Given an input *x* which has an incorrect model prediction. Denote ${\textbf{b}}:= \{{\mathbf {b}}_{k}\}_{k\in [K]}$, ${\mathbf {b}}_{k}:= \left( \ldots ~b_{k, a_{k}}~\ldots \right) _{a_{k}\in {\mathcal {A}}_{k}}^{\top }$, and $\Pr _{{\textbf{b}}}(Y=y \mid X=x):= \sum _{a_{1:K}} \Pr _{w}\left( Y=y \mid A_{1:K}=a_{1:K}\right) \cdot \prod _{k=1}^{K} b_{k, a_{k}}$. The counterfactual explanation for the ground-truth label *y* is a set of probability vectors ${\textbf{b}}$ that maximizes $\Pr _{{\textbf{b}}}(Y=y \mid X=x)$, i.e., the solution to the following optimization problem,

4 $$ \max _{{\textbf{b}}} {\operatorname {Pr}}_{{\textbf{b}}}(Y=y \mid X=x), \quad {\text {s.t.}} ~\sum _{a_{k}\in {\mathcal {A}}_{k}} b_{k, a_{k}} = 1~(0 \leqslant b_{k, a_{k}} \leqslant 1),\quad \forall k \in [K]. $$

We adopt the projected gradient ascent algorithm to generate the CEs, which is detailed in Algorithm 1.

**Algorithm 1:**
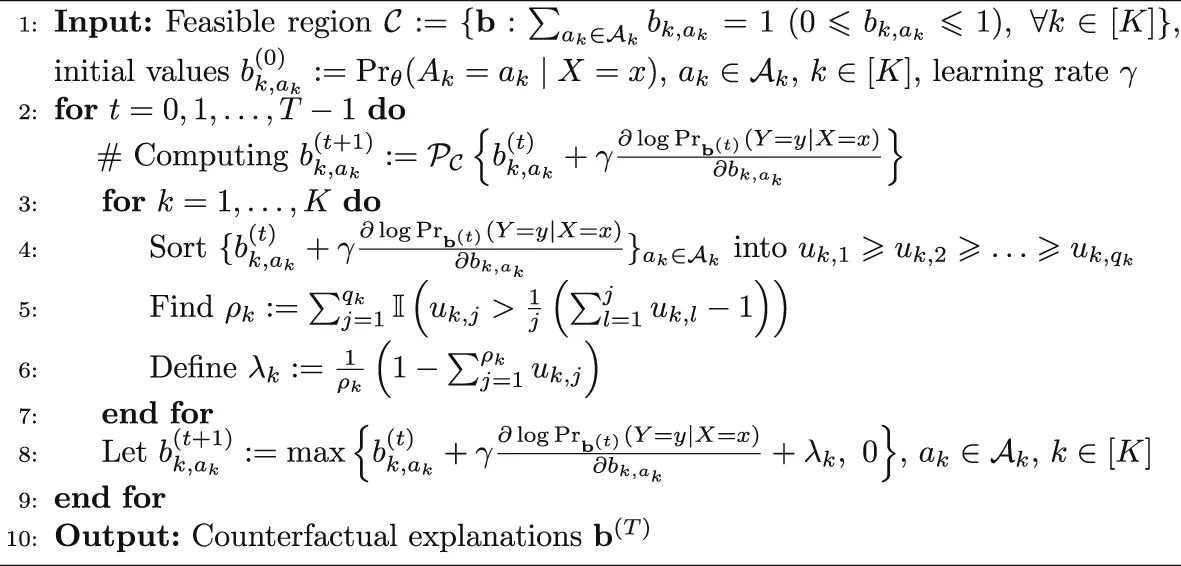
Generation of Counterfactual Explanations. The projection process is based on Wang and Carreira-Perpiñán (2013).

CEs reveal the model’s inner workings by identifying the attribute recognition changes required to correct an incorrect prediction. Similar to MPEs, these explanations deliver attribute-level insights into the model’s decision-making process, thereby improving interpretability.

Next, we introduce a metric to evaluate the effectiveness of CEs in correcting the model predictions.

#### Definition 4

(*Correction Rate*) The correction rate is defined as the proportion of initially incorrectly predicted samples that are corrected by CEs among all initially incorrectly predicted samples.

A high correction rate signifies that the generated CEs effectively correct model predictions by adjusting the attribute recognition results.

## Experiments

### Experimental Setup

***Datasets***

We evaluate the model performance on a variety of benchmark datasets. **(1) MNIST-Addition:** We derive this dataset from the original MNIST dataset (Lecun et al., 1998) by following the general preprocessing steps and procedures detailed in (Manhaeve et al., 2018). Each MNIST-Addition sample consists of two images randomly selected from the original MNIST. The digits in these images, ranging from 0 to 9, represent two attributes, with their sum serving as the class label. A total of 35,000 samples are created for MNIST-Addition. **(2) GTSRB:** GTSRB (Stallkamp et al., 2012) is a dataset comprising 39,209 images of German traffic signs, with class labels indicating the type of signs. Additionally, we annotate each sample with four attributes: “color”, “shape”, “symbol”, and “text”. The values for these attributes are detailed in Appendix 4. **(3) CelebA:** CelebA (Liu et al., 2015) consists of 202,599 celebrity face images annotated with 40 binary concepts. Here, we select the 8 most balanced binary concepts^2^ and group them into 5 attributes: “mouth”, “face”, “cosmetic”, “hair”, and “appearance”. Following Zarlenga et al. (2022), each unique combination of concept values is treated as a group. To balance the dataset and increase its complexity, we rank these groups by the number of images they contain and pair them strategically: the group with the most images is merged with the one with the fewest, the second most with the second fewest, and so on. The above strategy results in 127 total classes. **(4) AwA2:** AwA2 (Xian et al., 2018) contains 37,322 images of 50 types of animals, each annotated with 85 binary concepts. Certain concepts, such as those describing non-visual properties (e.g., “fast”, “domestic”) or indistinctive features (e.g., “chewteeth”), or those representing background information (e.g., “desert” and “forest”), are excluded. After the exclusion, 29 concepts remain, which are then grouped into 4 attributes: “color”, “surface”, “body”, and “limb”. The specific values for these attributes are provided in Appendix 4. For all datasets, we split the samples into training, validation, and testing sets by a ratio of 8:1:1.

***Baselines***

We select CBM (Koh et al., 2020) and several representative variants as baselines. Specifically, we include Hybrid CBM (Mahinpei et al., 2021), which extends CBM by incorporating unsupervised neurons into the concept bottleneck layer. We refer to the Hybrid CBM trained with supervision only on class labels as the *Class-Supervised Hybrid CBM*, and the one trained with supervision on both concepts and classes as the *Concept-and-Class-Supervised Hybrid CBM*. We also include CEM (Zarlenga et al., 2022), a method that uses high-dimensional concept embeddings as the bottleneck instead of concept probabilities, and DCR (Barbiero et al., 2023), which introduces a deep concept reasoner as the task predictor rather than relying on a simple linear layer. Additionally, we train an end-to-end DNN (He et al., 2016) as an additional baseline. It is important to note that Hybrid CBM, CEM, and the end-to-end DNN are not interpretable, as (some of) their components are not explicitly understandable by humans, even though they may achieve competitive performance on downstream tasks. A comparison of model properties is summarized in Table 1. Detailed descriptions of model architectures and training details are deferred to Appendix 3.

**Table 1:** Model properties

Model	Interpretability	Data-driven rules	Human-predefined rules	Theoretical guarantee
CBM (Koh et al., 2020)	✓	✗	✗	✗
Hybrid CBM (Mahinpei et al., 2021)	✗	✗	✗	✗
CEM (Zarlenga et al., 2022)	✗	✗	✗	✗
DCR (Barbiero et al., 2023)	✓	✓	✗	✗
NPC (ours)	✓	✓	✓	✓

“Interpretability” indicates whether the outputs produced by a concept/attribute recognition model are interpretable and whether humans can interpret the final decisions using these outputs. “Data-Driven Rules” denotes whether a model can incorporate logical rules learned from data. “Human-Predefined Rules” specifies whether a model can integrate logical rules predefined by humans. “Theoretical Guarantee” indicates whether a model provides a theoretical guarantee on the relationship between the performance of the overall model and that of its individual components.

***Evaluation Metrics***

Considering the compositional nature of NPCs, we introduce distinct evaluation metrics for the various modules as well as the overall model. **(1) Attribute Recognition Model:** We employ two metrics for evaluating the attribute recognition model. First, we propose the *mean total variation (TV) distance* between the output probability vectors from the attribute recognition model and the corresponding ground-truth probability vectors. The metric is defined as: $\frac{1}{K}\sum _{k}\frac{1}{|D_{\text {test}}|}\sum _{x \in D_{\text {test}}} d_{\text {TV}}(f_{k}(x;\theta ), g_{k}(x))$, where a smaller distance indicates better performance. For baseline models producing concept probabilities, we group the outputs into attributes and process them as probability vectors. Second, we adapt the *mean concept accuracy* metric used in (Zarlenga et al., 2022) for NPCs. For an input *x* with $n_{k}$ ground-truth values for the attribute $A_{k}$, we identify the top-$n_{k}$ values in $f_{k}(x; \theta )$ and set them to one while the rest are set to zero. We then calculate the accuracy by comparing these processed outputs with the ground-truth concept labels. **(2) Circuit-Based Task Predictor:** We use the *mean likelihood* to measure the performance of circuit-based task predictors, which is given by: $\frac{1}{|{\bar{D}}_{\text {test}}|}\sum _{(y, a_{1:K}) \in {\bar{D}}_{\text {test}}} \Pr _{w}(Y=y, A_{1:K}=a_{1:K})$. A larger mean likelihood suggests that the circuit models the joint distribution more accurately. **(3) Overall Model:** We adopt the *standard classification accuracy* as the evaluation metric for the overall model.

### NPCs vs. Baselines

We compare NPCs against six baseline models across four benchmark datasets, with the results summarized in Table 2. Specifically, we refer to the NPC whose circuit was learned using the data-driven approach as “NPC(Data)” and the NPC whose circuit was constructed using the knowledge-injected approach as “NPC(Knowledge)”.

As shown in Table 2, among all baselines trained with concept supervision (i.e., CBM, CC-Hybrid-CBM, CEM, and DCR), CC-Hybrid-CBM achieves the best performance on the MNIST-Addition and GTSRB datasets, showing the benefit of incorporating unsupervised neurons into the concept bottleneck layer. Meanwhile, CEM attains the highest accuracy on the CelebA and AwA2 datasets, demonstrating the advantage of using high-dimensional concept embeddings. These results indicate that higher task performance can often be obtained at the expense of reduced interpretability.

In contrast, NPCs achieve superior performance without introducing any uninterpretable components. Specifically, compared with the baselines above, NPC(Knowledge) performs best on the MNIST-Addition and GTSRB datasets, while NPC(Data) leads on the CelebA and AwA2 datasets. These findings underscore the effectiveness of NPCs in generating accurate class predictions based on the predicted concept distributions.

Remarkably, NPCs remain competitive even when compared with the end-to-end black-box baselines (i.e., DNN and C-Hybrid-CBM). In particular, they surpass these baselines in classification accuracy on the MNIST-Addition and GTSRB datasets. However, small performance gaps remain between the DNN and NPCs on more complex datasets such as CelebA and AwA2. Overall, these findings demonstrate that, while there is still room for improvement relative to black-box baselines, NPCs strike a compelling balance between interpretability and task performance. These results highlight the promising potential of interpretable models.

**Table 2:** Classification accuracy of NPCs and six baseline models on four benchmark datasets over five random seeds

Model	MNIST-Add (%)	GTSRB (%)	CelebA (%)	AwA2 (%)
${\text {DNN}}^{*}$	*99.057 ± 0.08*	${\underline{99.939}} \pm 0.04$	${\mathbf {36.963}} \pm 0.72$	**93.35** ± 0.17
C-Hybrid-CBM*	*98.126 ± 0.09*	*99.746 ± 0.06*	*21.404 ± 0.41*	87.217 ± 0.30
CBM	*98.606 ± 0.03*	*99.810 ± 0.04*	*16.552 ± 0.87*	82.286 ± 0.47
CC-Hybrid-CBM*	*98.796 ± 0.08*	*99.819 ± 0.08*	*17.649 ± 0.46*	83.398 ± 0.52
CEM*	*98.686 ± 0.13*	*99.766 ± 0.03*	*28.164 ± 0.55*	84.191 ± 0.58
DCR	*93.105 ± 1.23*	*98.034 ± 1.01*	*16.845 ± 2.09*	69.325 ± 2.46
NPC (data)	${\underline{99.171}} \pm 0.11$	*99.888 ± 0.08*	${\underline{33.739}} \pm 0.90$	87.281 ± 0.39
NPC (knowledge)	${\mathbf {99.189}} \pm 0.08$	${\mathbf {99.944}} \pm 0.04$	*31.727 ± 0.51*	68.519 ± 3.54

C-Hybrid-CBM and CC-Hybrid-CBM refer to Class-Supervised Hybrid CBM and Concept-and-Class-Supervised Hybrid CBM, respectively. “*” Denotes uninterpretable models. DNN and C-Hybrid-CBM are baselines trained without concept supervision. The best results for each dataset are highlighted in bold, while the second-best results are underlined.

### Ablation Studies

In this section, we delve into NPCs from additional perspectives. Specifically, we will analyze the integration of attributes, the influence of attribute selections, the effects of various approaches to constructing the task predictor, the impact of joint optimization, and lastly, the effect of interventions.

#### Attributes vs. Concepts

Unlike existing concept-based models that utilize individual binary concepts (e.g., “red color”, “yellow color”), NPCs focus on concept groups, i.e., attributes (e.g., “color”). Here, we aim to explore *the benefits of using attributes compared to individual concepts*. To this end, we replace the concept recognition model in CBM (Koh et al., 2020) with an attribute recognition model, resulting in a new model called the Attribute Bottleneck Model (ABM). ABM comprises an attribute recognition model and a linear layer serving as the task predictor. We employ the training loss from CBM to train ABM, replacing the concept loss with the attribute loss as defined in Eq. (2). The performance comparison between CBM and ABM is presented in Table 3.

**Table 3:** Comparison between CBM and ABM across four benchmark datasets in terms of mean TV distance and mean concept accuracy

Metric	Model	MNIST-Add	GTSRB	CelebA	AwA2
Mean TV Distance	CBM	0.0080	0.0023	**0.1744**	**0.0514**
	ABM	**0.0058**	**0.0007**	0.1759	0.0775
Mean Concept Accuracy	CBM	98.64%	99.80%	32.52%	82.21%
	ABM	**98.99%**	**99.84%**	**45.00%**	**89.69%**

For TV distance, lower values are better. For accuracy, higher values are better. The better values are in bold.

The results in Table 3 show that, in terms of the mean TV distance, ABM outperforms CBM on the MNIST-Addition and GTSRB datasets, albeit exhibiting slightly worse performance on the CelebA and AwA2 datasets. On the other hand, ABM consistently surpasses CBM in mean concept accuracy across all datasets. These findings underscore the effectiveness of the attribute recognition model, indicating that attributes capture more nuanced information. In particular, we hypothesize that inherent relationships may exist both within the values of any single attribute and across different attributes. Therefore, treating all values as independent concepts neglects these interdependencies, potentially leading to inferior performance. Overall, these results suggest the benefits of utilizing attributes in preserving relational constraints within predictions and thus improving model performance.

#### Impact of Attribute Selection

During inference, an NPC utilizes sufficient attributes to produce final predictions. Here, we aim to investigate the following questions: *How does the exclusion of one particular attribute during inference impact the performance of NPC on downstream tasks?* *How does the exclusion of different attributes vary the impact?*

**Fig. 3:**
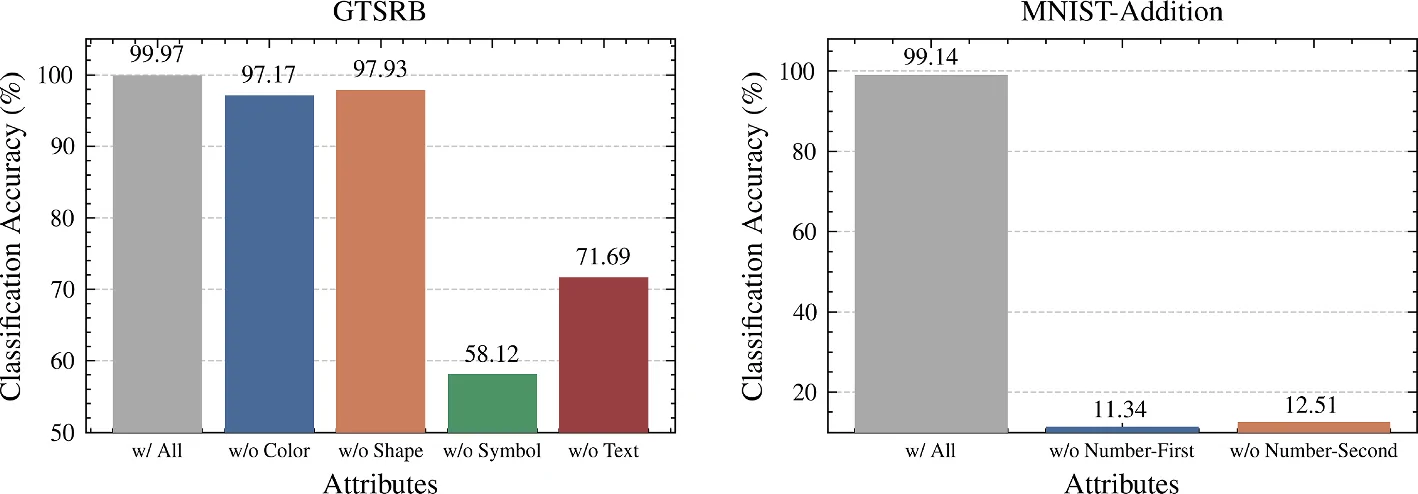
NPC classification accuracy on the GTSRB and MNIST-Addition datasets with attribute exclusions. The gray bars indicate inference with all attributes, while non-gray bars indicate inference with one particular attribute excluded.

Assuming the excluded attribute is $A_{1}$, the predicted score for the class *y* would become $\sum _{a_{2:K}}\Pr _{w}(Y=y\mid A_{2:K}=a_{2:K})\cdot \prod _{k=2}^{K}\Pr _{\theta} (A_{k}=a_{k}\mid X)$. We apply this inference process on the GTSRB dataset and the resulting task performance is presented in Fig. 3 (Left).

We observe that, when NPC has one fewer attribute at its disposal during inference, the classification accuracy decreases, due to the fact that the assumption of sufficient attributes (Assumption 1) no longer holds in this scenario, and that the equality in Eq. (1) was violated. Therefore, the formula above does not correctly represent $\Pr _{\theta ,w}(Y=y\mid X)$. As a result, relying on the above formula for inference can adversely affect the predictions.

On the other hand, we observe that excluding different attributes can lead to varying impacts on the task performance. Specifically, excluding the “color” or “shape” attributes results in only a slight drop in accuracy, whereas excluding the “symbol” or “text” attributes leads to a significant decline. We accredit such discrepancy to the distinct properties of these attributes. More specifically, attributes such as “color” and “shape” are generally not decisive, meaning that they do not decisively determine the final class, and that their absence can be compensated by leveraging information from the other attributes. For instance, even without having any indication of “red” or “octagon”, it is still possible to infer an input representing a stop sign so long as the “text” attribute indicates “stop”. Thus, excluding the non-decisive attributes has minimal impact on the performance. In contrast, “symbol” and “text” attributes are decisive for many instances and thus are crucial for distinguishing between certain classes. For example, without the “text” attribute, it then becomes impossible to differentiate speed limit signs indicating different speeds. Similarly, without the “symbol” attribute, distinguishing between a left-curve and a right-curve sign is no longer feasible. Hence, excluding decisive attributes can severely impair predictions.

For the MNIST-Addition dataset, Fig. 3 (Right) demonstrates similar findings. In particular, as both attributes are essential for determining the final class, i.e., the sum, the removal of either attribute results in a substantial performance decline.

In summary, utilizing insufficient attributes compromises NPC’s performance on downstream tasks, and the impact of excluding different attributes varies depending on the properties of the attributes themselves.

#### Impact of Task Predictor Construction Approaches

In Section 3.2.2, we introduce two distinct approaches for constructing probabilistic circuits: the data-driven approach and the knowledge-injected approach. Here, our objective is to investigate the impact of these construction methods. Specifically, we aim to address the following questions: *Which approach produces a circuit that better captures the data distribution? Which approach produces a circuit that performs more effectively as a task predictor?* We start by comparing the mean likelihood of the two circuits. Then, we examine the classification accuracy of the overall models consisting of a well-trained attribute recognition model together with either circuit (data driven or knowledge injected). The results are summarized in Table 4.

**Table 4:** Performance comparison between circuits constructed using data-driven and knowledge-injected approaches, along with the classification accuracy of overall models combining either circuit with a well-trained attribute recognition model

Metric	Architecture	MNIST-Add	GTSRB	CelebA	AwA2
Mean Likelihood	Circuit (Data)	**1.010e−2**	**3.663e−02**	2.082e−02	**1.263e−04**
	Circuit (Knowledge)	1.007e−2	**3.663e−02**	**2.091e−02**	1.255e−04
Accuracy	Model (Data)	**99.17%**	**99.85%**	**32.74%**	**68.52%**
	Model (Knowledge)	**99.17%**	**99.85%**	31.56%	23.47%

The better results are in bold.

In terms of classification accuracy, the models combining the different circuits exhibit similar performance on MNIST-Addition, GTSRB, and CelebA datasets. Such similarity suggests that the two-layered circuit constructed using the knowledge-injected approach is sufficient to provide accurate relational information among attributes and classes for simpler datasets.

In contrast, for the more complex AwA2 dataset, Model (Data) outperforms Model (Knowledge) by a wide margin due to the presence of multi-valued attributes in AwA2, which results in a large number of valid combinations of attribute values. Consequently, each combination may correspond to a very small joint probability. Under these circumstances, even a slight difference in mean likelihood may induce a significant change in the circuit’s ability to capture the data distribution. For instance, even a marginally lower mean likelihood might indicate the failure of the circuit to capture the joint probabilities of a large quantity of combinations. As shown in Table 4, the mean likelihood of Circuit (Knowledge) is slightly lower than that of Circuit (Data) for the AwA2 dataset, suggesting that, in this case, the knowledge-injected circuit might have failed to capture sufficient nuanced relationships among attributes and classes, while the data-driven circuit is better suited for representing the joint distribution of the data, thus leading to a better performance on the downstream task.

#### Impact of Structure Learning

In Section 3.2.2, we describe how to learn the structure of a probabilistic circuit from data and how to manually construct it to encode domain knowledge. Here, we take a step further and investigate *how circuit structure learning affects the overall performance of NPC.*

**Fig. 4:**
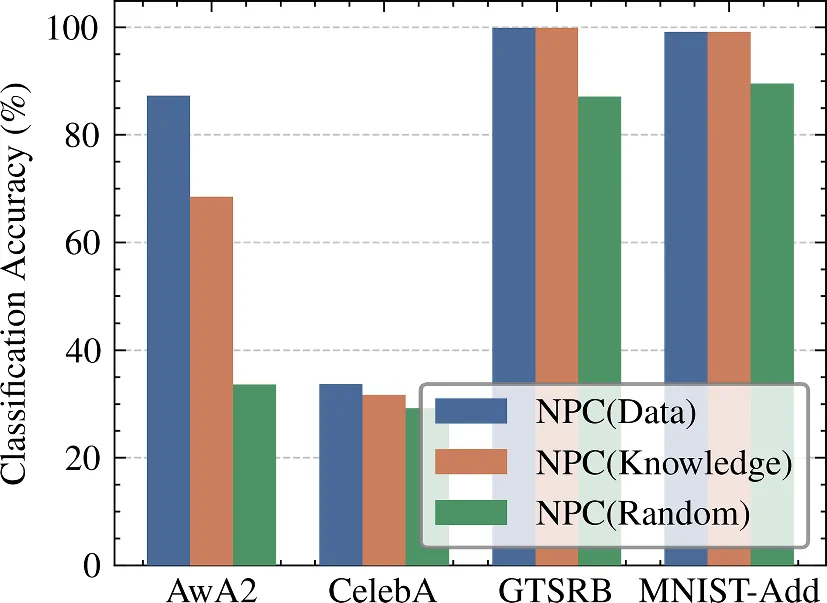
Classification accuracy of NPC models with different circuit structures across four benchmark datasets. NPC(Data) uses a structure learned from data via LearnSPN (Gens & Domingos, 2013). NPC(Knowledge) employs a manually constructed structure that encodes domain knowledge. NPC(Random) adopts a randomly generated structure that satisfies smoothness and decomposability.

To this end, we initialize the probabilistic circuit in the second stage with a random structure that satisfies smoothness and decomposability. This variant, known as RAT-SPN (Peharz et al., 2019), starts from a random region graph and populates it with arrays of sum and product nodes to form a random structure for the circuit. Fig. 4 compares the classification accuracy of NPC models with different circuit structures across four benchmark datasets.

The results show that, among the three NPC models, NPC(Random) consistently performs the worst. It can be attributed to the structural design. The data-driven approach enables the circuit to capture context-specific independencies that enhance expressiveness, while the knowledge-injected approach ensures that the circuit learns distributions consistent with domain constraints. In contrast, a randomly generated structure lacks these properties, which reduces its expressiveness and leads to degraded performance. Overall, these findings highlight the importance of circuit structure learning. In particular, a well-learned or knowledge-guided structure enhances the expressiveness of the probabilistic circuit, thereby leading to improved performance of the NPC.

#### Impact of Joint Optimization

**Fig. 5:**
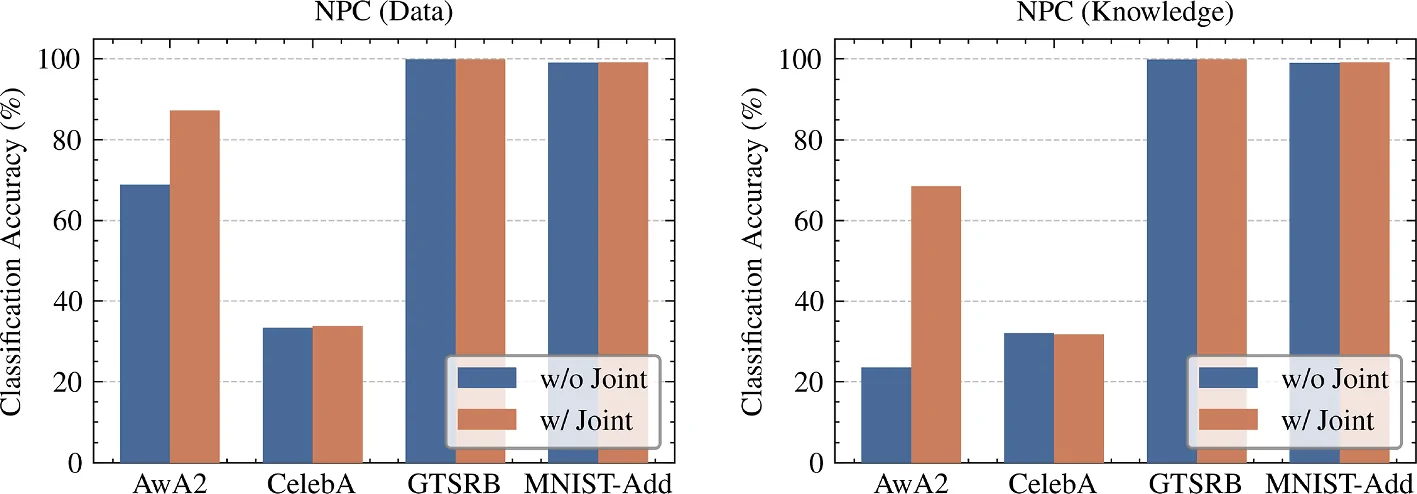
Classification accuracy of NPC(Data) and NPC(Knowledge) on the four benchmark datasets. The blue bars indicate performance prior to joint optimization, while the orange bars illustrate performance after applying joint optimization.

When training the NPCs, we adopt a three-stage training algorithm, where we first independently train the attribute recognition model and the task predictor, followed by a joint optimization for the overall model. Here, we aim to investigate *how the third stage, i. e., joint optimization, affects the performance of NPCs*. To this end, we compare the performance of NPCs before and after applying the joint optimization. The comparison is illustrated in Fig. 5.

In general, joint optimization enhances model performance across the various datasets. Specifically, for the AwA2 dataset, joint optimization leads to substantial improvements for both NPC(Data) and NPC(Knowledge), demonstrating its effectiveness. In contrast, for the CelebA dataset, the performance stays roughly the same after applying joint optimization, with a marginal increase in performance for NPC(Data) and a marginal decrease in performance only for NPC(Knowledge). Nevertheless, slight performance improvements are generally observed on the GTSRB and MNIST-Addition datasets for both NPCs. Overall, these results suggest that joint optimization provides additional benefits for NPCs in terms of downstream tasks. In particular, for datasets with superior performance after the initial training, joint optimization provides additional but modest gains. In contrast, for datasets with moderate performance after the initial training, joint optimization plays a pivotal role in delivering significant performance improvements.

#### Impact of Interventions

One special property of concept-bottleneck models is that they permit interventions. Specifically, they allow human experts to intervene on a concept by correcting its predicted value to the ground truth. The performance under interventions reflects how responsive a concept bottleneck model is and indicates the extent to which its behavior aligns with human expectations. In this section, we investigate *the responsiveness of NPC models to interventions*.

To this end, we perform interventions on both NPC models before and after joint optimization. For each NPC model, we consider two types of probabilistic circuits: those learned from data and those manually constructed based on domain knowledge. The interventions are conducted on all four datasets. Taking the GTSRB dataset as an example, which contains four attributes, we intervene on one, two, three, and all four attributes, respectively. When intervening on a single attribute, we intervene on “color”, “shape”, “symbol”, and “text”, respectively, and report the classification accuracy, mean TV distance, and mean concept accuracy, averaged over these four cases. When intervening on two attributes, we intervene on all $$\left( {\begin{array}{c}4\\ 2\end{array}}\right) $$ possible combinations of attributes, respectively, and report the same metrics averaged across these $$\left( {\begin{array}{c}4\\ 2\end{array}}\right) $$ cases. The same procedure applies for interventions on three and four attributes. Interventions are implemented by replacing the probability vector predicted by the attribute recognition model for a specific attribute (e.g., “color”) with the corresponding ground-truth label vector.

**Fig. 6:**
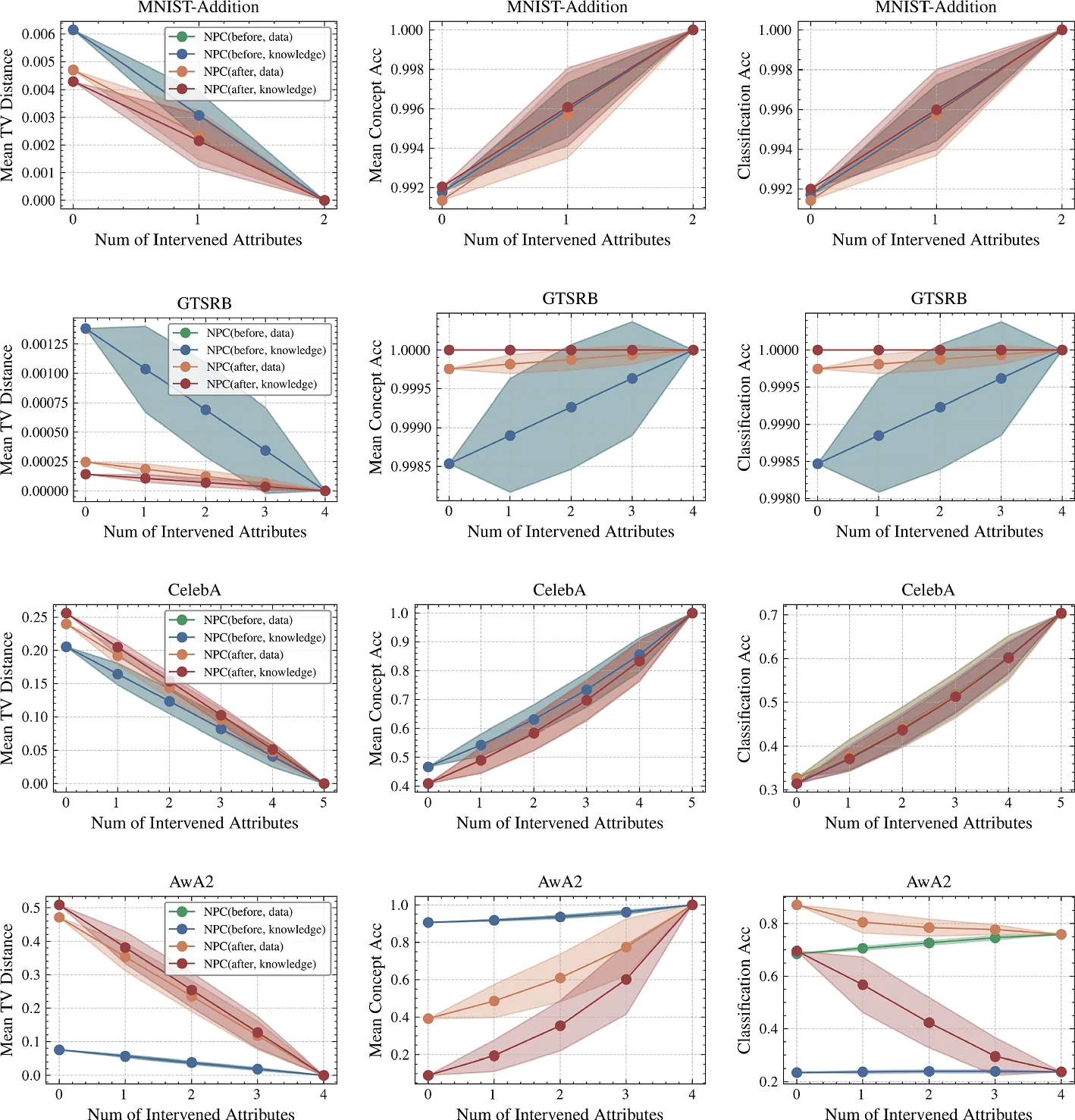
The mean TV distance **(left)**, mean concept accuracy **(middle)**, and classification accuracy **(right)** of various NPC models (before and after joint optimization, with probabilistic circuits either learned from data or manually constructed from knowledge) under different numbers of intervened attributes on the MNIST-Addition **(Row 1)**, GTSRB **(Row 2)**, CelebA **(Row 3)**, and AwA2 **(Row 4)** datasets. The solid lines denote the average values, and the shaded regions indicate the standard deviations, both computed over all intervention cases with the same number of intervened attributes.

Figure 6 illustrates how the mean TV distance, mean concept accuracy, and classification accuracy vary with the number of intervened attributes. We separately analyze NPC models before and after joint optimization.

For NPC models before joint optimization (regardless of whether the probabilistic circuit is learned or manually constructed), we observe that as more attributes are intervened, the mean TV distance decreases and the mean concept accuracy increases. In particular, when all attributes are intervened, the mean TV distance reaches 0 and the mean concept accuracy reaches *100%*. Remarkably, the classification accuracy also improves as the number of intervened attributes increases. These results demonstrate that the decision-making process of these models aligns with human expectations.

For NPC models after joint optimization, similarly, the mean TV distance decreases and the mean concept accuracy increases as more attributes are intervened. Moreover, for most datasets, the classification accuracy also improves with an increasing number of intervened attributes. However, this trend does not hold for the AwA2. We hypothesize that on this more complex dataset, the interpretability of the NPC model is compromised during joint optimization, where supervision is provided only through class labels, leading to reduced benefit from interventions. The gaps in mean concept accuracy between models before and after joint optimization when no attributes are intervened (Row 4, middle) support this hypothesis. Nevertheless, interventions in jointly trained NPC models remain aligned with human expectations across most datasets. In particular, when all attributes are intervened, the classification accuracy reaches *100%* on GTSRB and MNIST-Addition and exceeds *70%* on CelebA.

Overall, the responses of NPC models to interventions are largely consistent with human expectations.

### Model Explanations

In this section, we explore two types of explanations and provide examples to illustrate how the explanations facilitate the human’s understanding of NPC’s inner workings and interpret the model’s behavior.

#### Most Probable Explanations

**Fig. 7:**
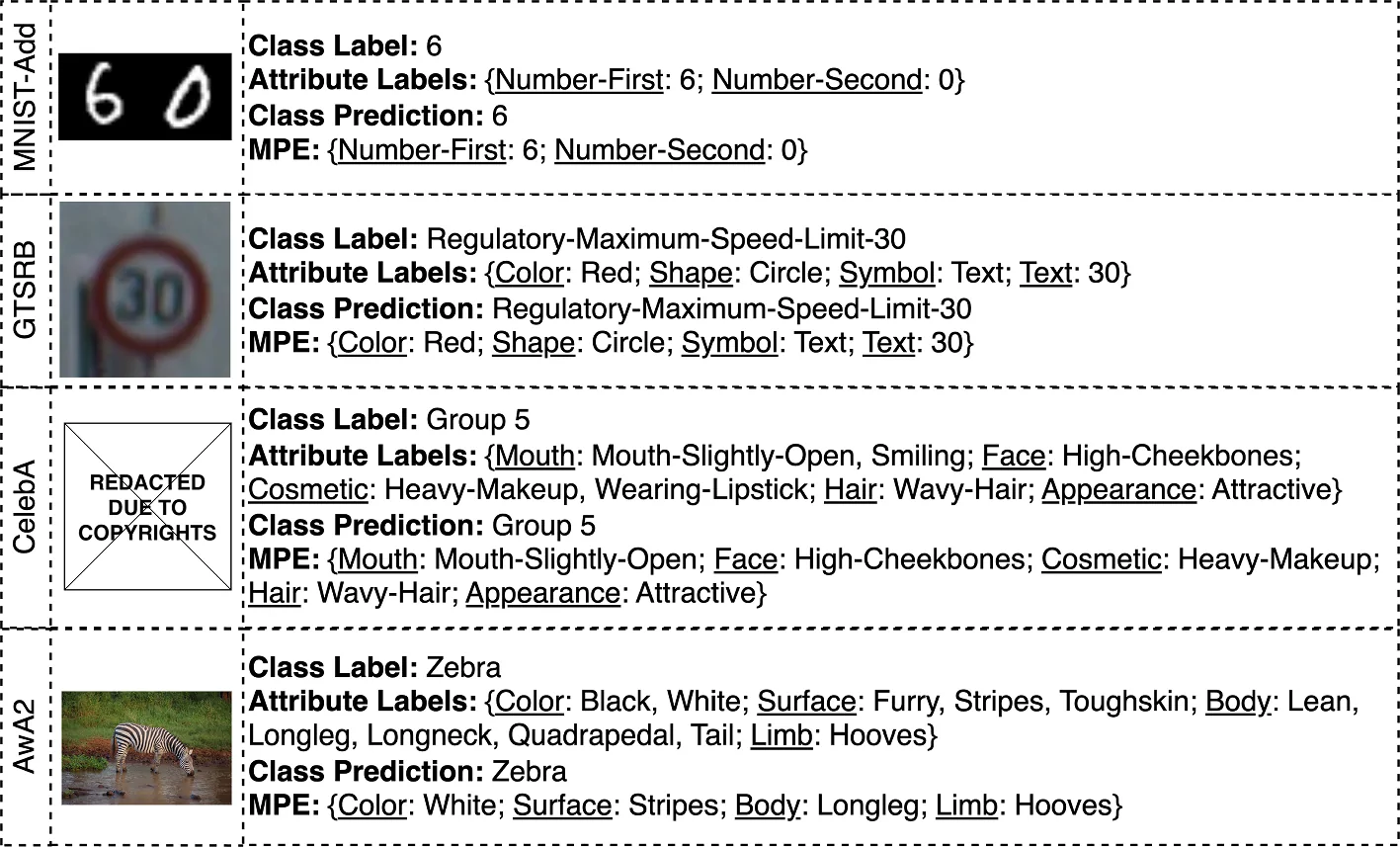
Examples from the four benchmark datasets. Each example includes an image, the ground-truth class label, the ground-truth attribute labels, the **NPC(Data)** class prediction, and, lastly, the corresponding MPE. These examples illustrate MPEs that align with the ground-truth attribute labels. The CelebA image is redacted in compliance with its terms of use.

**Fig. 8:**
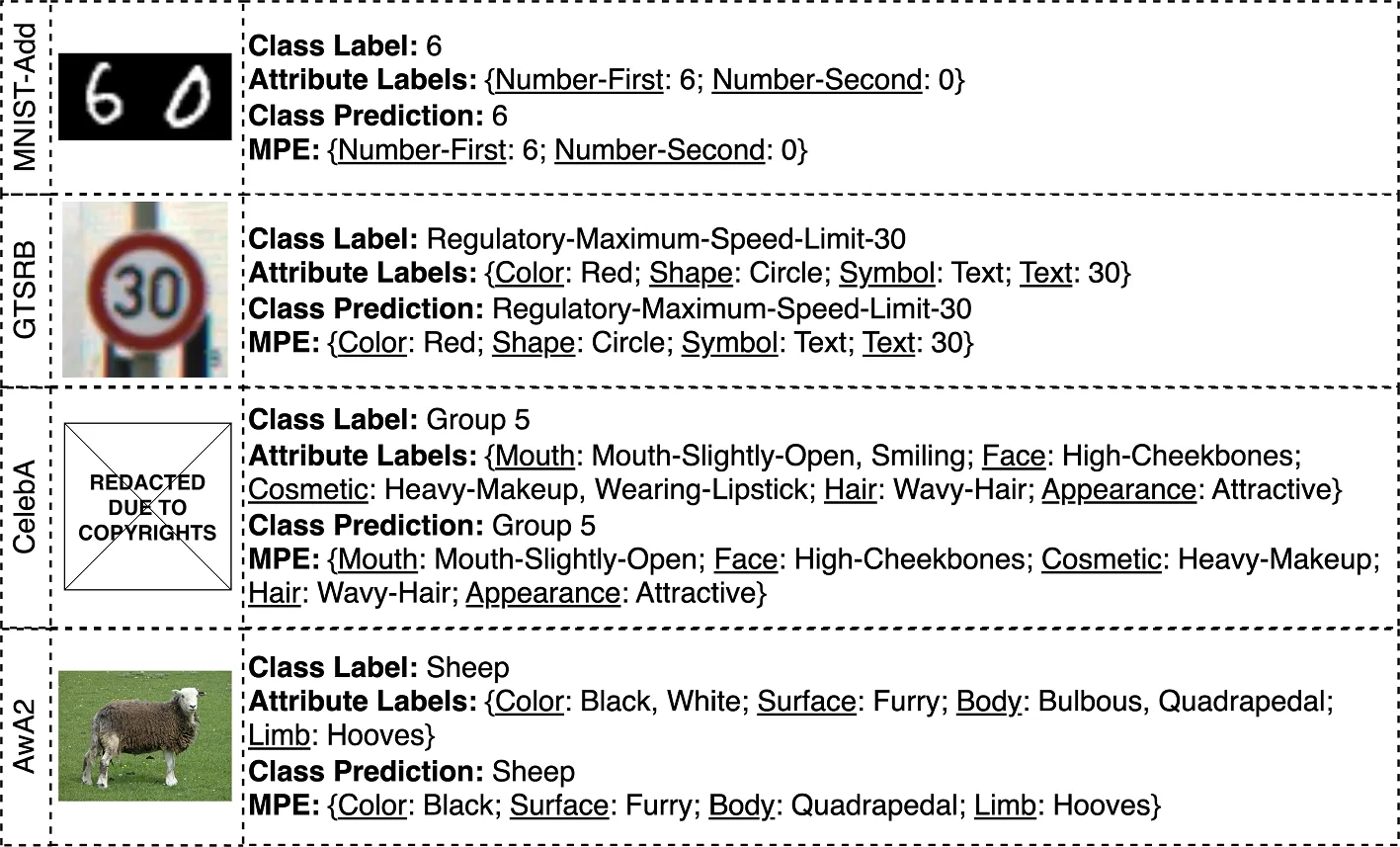
Examples from the four benchmark datasets. Each example includes an image, the ground-truth class label, the ground-truth attribute labels, the **NPC(Knowledge)** class prediction, and, lastly, the corresponding MPE. These examples illustrate MPEs that align with the ground-truth attribute labels. The CelebA image is redacted in compliance with its terms of use.

Figures 7 and 8 present examples for NPC(Data) and NPC(Knowledge), respectively, across the four benchmark datasets. Specifically, each example comprises an image, the ground-truth labels for the class and attributes, the class predicted by NPC(Data), and, lastly, the corresponding MPE which accounts for the attribute assignment that contributes most significantly to the prediction. In these instances, NPC(Data) provides correct class predictions, and the MPEs are aligned with the ground-truth attribute labels. For instance, the MPE for the example from GTSRB is {Color: Red; Shape: Circle; Symbol: Text; Text: 30}, which precisely matches the ground-truth attribute labels. Such alignment between MPEs and attribute labels indicates that the model employs human-like reasoning and makes reliable decisions.

**Fig. 9:**
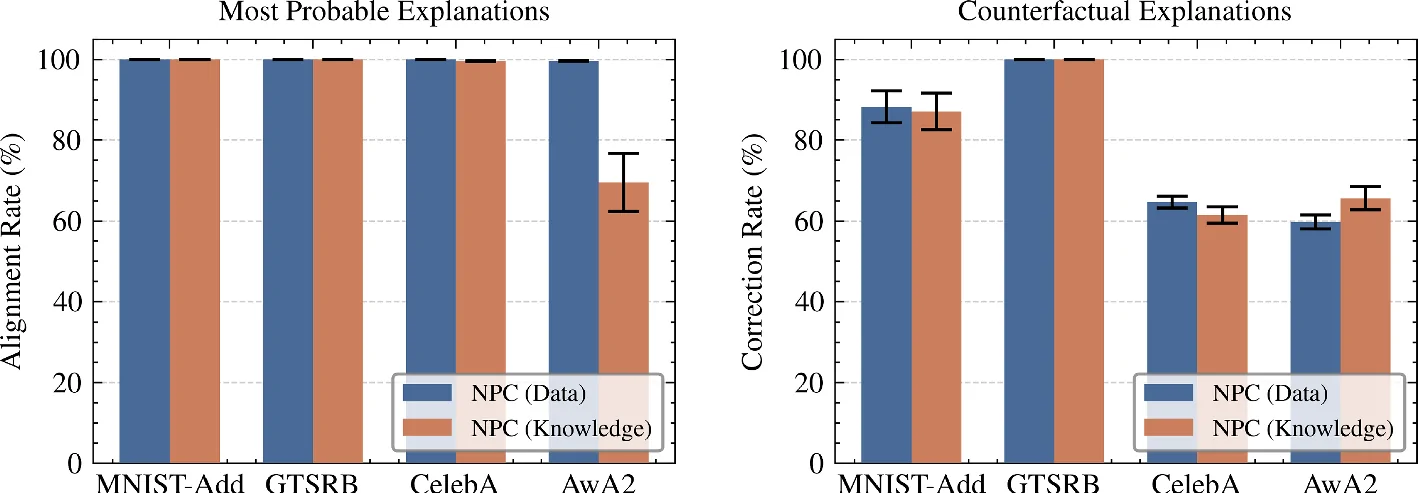
MPE alignment rate and CE correction rate for NPCs across the four benchmark datasets. The blue bars represent NPC(Data), while the orange bars represent NPC(Knowledge). Error bars indicate the standard deviation over five random seeds.

The MPEs alignment rates are shown in Fig. 9 (Left). We observe that NPC(Knowledge) exhibits a relatively low alignment rate on the AwA2 dataset, suggesting that the ground-truth attribute assignment does not contribute the most to the correct predictions for certain instances. Such misalignment could be caused by the knowledge-injected approach failing to capture the relatively more complex conditional dependencies between classes and attributes for this particular dataset, thereby affecting the model’s prediction process. On the other hand, the MPEs alignment rates in the other scenarios are close to 100%, indicating that when it makes correct predictions, the model predominantly relies on attribute assignments matching the ground truths. Therefore, the model is considered reliable as its prediction process properly aligns with the human’s decision-making process.

**Fig. 10:**
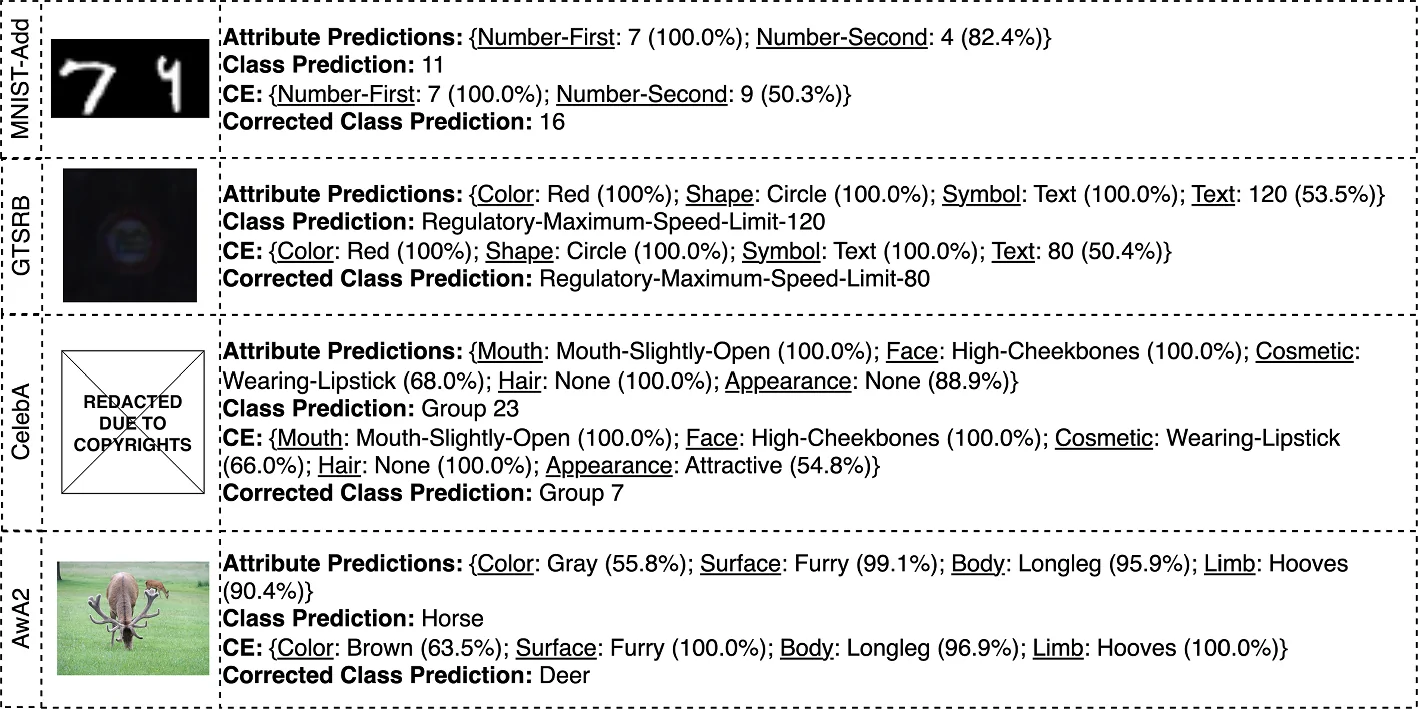
Examples from the four benchmark datasets. Each example includes an image, the **NPC(Data)** attribute and class predictions, the corresponding CE, and the class prediction corrected by CE. These examples focus on CEs that successfully correct the class predictions. For simplicity, both attribute predictions and CEs display only the most confident value for each attribute. The CelebA image is redacted in compliance with its terms of use.

**Fig. 11:**
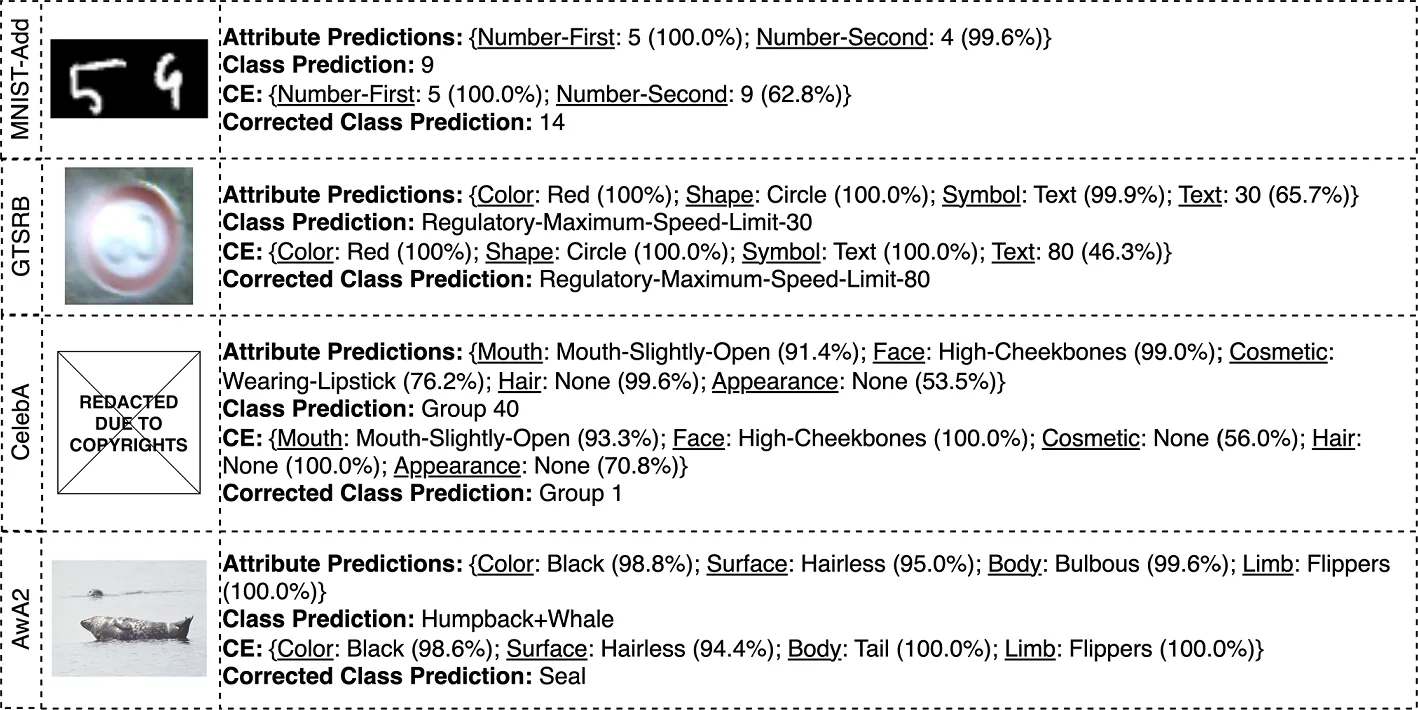
Examples from the four benchmark datasets. Each example includes an image, the **NPC(Knowledge)** attribute and class predictions, the corresponding CE, and the class prediction corrected by CE. These examples focus on CEs that successfully correct the class predictions. For simplicity, both attribute predictions and CEs display only the most confident value for each attribute. The CelebA image is redacted in compliance with its terms of use.

#### Counterfactual Explanations

Figures 10 and  11 illustrate examples for NPC(Data) and NPC(Knowledge), respectively, across the four benchmark datasets. Each sample consists of an image, the attribute and class predictions, the generated CE, and, lastly, the class prediction corrected by the CE. In these examples, NPC(Data) incorrectly predicts the class, while CEs effectively correct them by making minimal adjustments to the attribute predictions.

Take NPC(Data) as an example. As shown in Fig. 10, on the MNIST-Addition dataset, NPC(Data) initially predicts the “number-first” attribute as “7”, the “number-second” attribute as “4”, and the sum of these attributes (i.e., the class) as “11”. However, the prediction of the “number-second” attribute is incorrect since the ground-truth label is “9”. The generated CE indicates that if this attribute were instead predicted as “9”, even with a relatively low confidence of 50.3%, NPC(Data) would have correctly predicted the sum as “16”. These observations suggest that, on one hand, NPC(Data) follows a correct reasoning process, since both 7 + 4 = 11” and “7 + 9 = 16” are valid; on the other hand, only a low confidence in the correct attribute prediction is sufficient to yield the correct class prediction.

On the GTSRB dataset, NPC(Data) initially mispredicts the “text” attribute as “120”, while the remaining attributes are correctly predicted. Consequently, the model incorrectly classifies the sample as a “speed limit 120” sign. The generated CE shows that, if the “text” attribute were instead predicted as “80”, even with a confidence of only 50.4%, the model would have correctly classified the sample as a “speed limit 80” sign. Similar patterns are observed on the other datasets. Specifically, on the CelebA dataset, predicting the “appearance” attribute as “attractive” leads to the correct class label, while on the AwA2 dataset, predicting the “color” attribute as “brown” results in the correct classification. Together, these observations suggest there exist certain decisive attributes, and mispredicting them can severely impact the final decision, which aligns with the findings discussed in Section 5.3.2.

The CE correction rates are presented in Fig. 9 (Right). For simpler datasets like MNIST-Addition and GTSRB, the generated CEs exhibit high correction rates, indicating their effectiveness in correcting predictions. However, for more complex datasets such as CelebA and AwA2, the correction rates are lower, highlighting the limit of the efficacy of the generated CEs on complex datasets. Such a limit underscores the future need for more advanced algorithms for generating CEs that can correct misclassifications with excellent correction rates, even for complex datasets.

## Limitations and Discussion

In this section, we discuss the limitations of NPCs from multiple perspectives, highlighting potential future directions for improvement.

***Model Architecture***

Compared to end-to-end DNNs, NPCs offer superior interpretability by decomposing a model into semantically meaningful modules, enabling humans to combine module outputs to understand the final decisions. Nevertheless, the attribute recognition model itself remains a black box, and its opaque inner workings make it difficult to ensure that its outputs truly represent the probabilities for the various attributes. For instance, the model might learn spurious correlations and incorrectly map background features, instead of actual attributes, to outputs. Future work may focus on increasing the transparency within the attribute recognition model, thereby enhancing its interpretability.

***Structure of Probabilistic Circuits***

In NPC, the task predictor, implemented using a probabilistic circuit, is either learned using LearnSPN (Gens & Domingos, 2013) or manually constructed based on human-predefined rules. The circuit generated by LearnSPN, however, may contain an excessive number of nodes and edges, resulting in a slower inference. On the other hand, manually constructed circuits employ simpler structures with only two layers. While it may improve efficiency, such simplicity may limit the circuit’s expressiveness, potentially degrading its performance on complex datasets like AwA2. These results underscore the importance of designing a probabilistic circuit structure that achieves a balance between structural complexity and expressiveness.

One promising direction is to enrich node operations by incorporating more arithmetic operations. For instance, Peharz et al. (2020) vectorize circuit nodes and perform all product and sum operations on the same topological layer using a single monolithic einsum operation. Another direction is to build deeper structures, which typically have stronger expressiveness. To achieve this, several approaches improve upon the original LearnSPN (Gens & Domingos, 2013) framework by introducing different clustering (Butz et al., 2018; Krakovna & Looks, 2016; Liu & Luo, 2019; Vergari et al., 2015) and partitioning (Mauro et al., 2017) strategies. Finally, pruning redundant nodes can simplify the structure while preserving accuracy, as shown in the methods inspired by neural network pruning (Ventola et al., 2020) and self-attention mechanisms (Yu et al., 2021). Overall, these directions provide effective pathways to balance the structural complexity and expressiveness of probabilistic circuits.

***Assumption of Sufficient Attributes***

This is a common assumption in concept bottleneck models, where concepts are expected to fully determine the class label. However, because concept-level annotations are often scarce and costly, situations may arise where the available concepts are insufficient. Several strategies can be adopted to address this issue, such as using large language models (LLMs) to augment concept annotations, as explored in concept-annotation-free CBMs (Oikarinen et al., 2023; Yüksekgönül et al., 2023), or incorporating unsupervised neurons into the bottleneck, as demonstrated by Mahinpei et al. (2021). Beyond these general strategies, a solution naturally aligned with the NPC framework is to replace the probabilistic circuit with a conditional probabilistic circuit (Shao et al., 2022) that can compute $\Pr (Y|A_{1:K},X)$. This modification allows the NPC to leverage additional information from the input when attributes are insufficient.

***Assumption of Complete Information***

The complete information assumption states that the attributes are mutually independent given the input. While this assumption helps reduce computational complexity when computing $\Pr (A_{1:K}|X)$, recent studies have shown that it can limit model expressiveness (Krieken et al., 2024) and introduce spurious correlations, also known as reasoning shortcuts (Bortolotti et al., 2024; Marconato et al., 2023).

Suppose the underlying causal structure follows $X \rightarrow A_{1:K} \rightarrow Y$. If there now exists a confounder *Z* influencing both *X* and $A_{1:K}$, then this assumption may not hold. In such cases, even when the input *X* is observed, the attributes $A_{1:K}$ are no longer mutually independent because they share the influence of *Z*. For example, *X* denotes a patient’s overall health record, $A_{1:K}$ represents medical or lifestyle attributes such as smoking status, and *Y* indicates whether the patient has lung cancer. Suppose there exists an unobserved family factor *Z* that affects both the patient’s lifestyle behaviors and their genetic susceptibility to diseases. Then *Z* acts as a confounder for the $X \rightarrow A_{1:K}$ relationships, making the attributes correlated even when conditioning on *X*.

Recent work (Dominici et al., 2025) incorporates a causal concept graph into the concept-based model. The incorporated causal relationships could simplify the modeling of attribute distributions while avoiding the conditional independence assumption. The assumption can also be relaxed by partitioning the attributes based on conditional independence. Specifically, one can use independence tests to identify groups of attributes that are conditionally independent given the inputs, and then train an attribute recognition model in which each recognition head models the joint distribution over the variables within one group. This is similar to using a non-diagonal normal distribution (Vandenhirtz et al., 2024) to capture the attribute dependencies. In summary, these approaches can provide a better balance between computational efficiency and model expressiveness.

***Reducing Trade-Offs Between Interpretability and Task Performance***

In this paper, we show that, with the integration of attribute recognition and probabilistic circuit, NPC produces interpretable predictions for downstream tasks while achieving superior performance. Looking ahead, we believe that, by incorporating more fine-grained and diverse attributes that are semantically meaningful, along with a structure that reasons over these attributes using logical rules with increased complexity, we shall devise compositional model designs that further reduce the trade-offs between interpretability and performance of downstream tasks.

***Mitigating Potential Influence of Joint Optimization***

As discussed in Section 5.3.6, joint optimization can reduce concept accuracy and thereby compromise model interpretability. Such degradation in concept accuracy after joint optimization remains an open problem in neuro-symbolic learning. Recent studies have described this phenomenon as a reasoning shortcut (Marconato et al., 2023), where concept predictors tend to capture unintended correlations when concept-level supervision is absent. To address this issue, several mitigation strategies (Bortolotti et al., 2024; Krieken et al., 2025; Marconato et al., 2025, 2024, 2023) have been proposed, including incorporating explicit concept supervision, applying entropy regularization and smoothing to stabilize concept learning, introducing reconstruction and contrastive objectives to promote semantic consistency, and enforcing architectural disentanglement to prevent shortcut learning. These directions suggest promising ways to preserve interpretability while maintaining high task performance under joint optimization.

***Extending NPCs to Real-World Scenarios***

Extending NPCs to real-world scenarios presents several practical challenges and opportunities. First, training NPCs requires datasets with dense concept annotations; however, such datasets are rare in practice as collecting concept labels is both time-consuming and labor-intensive. Recent concept bottleneck models (Oikarinen et al., 2023; Yüksekgönül et al., 2023) leverage large language models to generate rich concept sets and employ multi-modal models to annotate concepts based on similarity in the embedding space. Similar concept annotation strategies can be adopted in NPCs to address the scarcity of concept annotations. Second, probabilistic circuits often struggle with practical scalability. The structural constraints required for tractable inference result in highly sparse and cluttered computational graphs which are hard to train. Einsum Networks (Peharz et al., 2020) improve this by reformulating the circuits using tensorized layers and einsum operations. Integrating this formulation into NPCs could enhance their scalability. Finally, the computational complexity of NPCs grows exponentially with the number of attributes, limiting their applicability when dealing with large attribute spaces. One potential direction to mitigate this issue is to employ tensor decomposition techniques or approximation-based methods from the neuro-symbolic learning literature (Krieken et al., 2025, 2023; Li et al., 2023; Verreet et al., 2024) to reduce the complexity. Overall, these directions highlight promising paths toward making NPCs more scalable and applicable to large-scale, real-world datasets.

## Related Work

In this section, we discuss several areas of research relevant to our proposed method.

***Concept Bottleneck Models and Variants***

Concept bottleneck models (CBMs) and their variants are a class of machine learning models that organize their decision-making process around high-level, human-understandable concepts, offering enhanced transparency. First introduced by Koh et al. (2020), CBMs decompose a black-box DNN into two modules: a concept recognition model, responsible for predicting various human-specified concepts, and a task predictor, which performs classifications on the predicted concepts.

Subsequent research has focused on improving these two modules. Zarlenga et al. (2022), Yeh et al. (2020), and Kazhdan et al. (2020) extend the concept recognition model by representing concepts as high-dimensional embeddings rather than simple probabilities. Additionally, Mahinpei et al. (2021), Sawada and Nakamura (2022), Sarkar et al. (2022), and Marconato et al. (2022) introduce unsupervised neurons into the bottleneck to enhance the model’s learning capacity. While improving the performance on downstream tasks, these extensions compromise interpretability, as the dimensions within concept embeddings and the unsupervised neurons lack explicit semantic meanings. In contrast, utilizing predicted concept probabilities gives better interpretability. On the other hand, there have been recent efforts to improve the interpretability of the task predictor. Instead of using a linear layer as the task predictor, several approaches (Barbiero et al., 2023; Ciravegna et al., 2023; Debot et al., 2024; Rodríguez et al., 2024) design new architectures to embed logical rules and enable classifications via reasoning. Barbiero et al. (2023) propose the Deep Concept Reasoner (DCR), which generates fuzzy logic rules using neural models on concept embeddings, and then evaluates these rules symbolically using the concept truth degrees. However, this architecture does not support the incorporation of human-defined rules.

Recently, Debot et al. (2024) introduce the Concept-based Memory Reasoner (CMR), which integrates a neural selection mechanism over a memory of learnable logic rules, followed by a symbolic evaluation of the selected rule. Unlike DCR, CMR learns logic rules within a memory that allows human-defined rules to be injected. This concurrent work shares similar functionality with NPC, as both can encode data-driven and human-defined rules, produce interpretable class predictions, and maintain strong expressiveness. However, the two differ in model architectures and inference processes. Specifically, in addition to the concept recognition model and task predictor, CMR includes a rule selector composed of neural networks and rule embeddings. The rule selector learns multiple rule distributions, selects the most likely rule from each, and evaluates the rule symbolically using the concept truth degrees. In contrast, the task predictor in NPC, i.e., the probabilistic circuit, compiles rules into its structure and learns the joint distribution over classes and attributes. It supports tractable and exact probabilistic inference, which NPC leverages to produce class predictions based on the attribute prediction scores.

***Probabilistic Circuits***

Probabilistic circuits (Sánchez-Cauce et al., 2022) are rooted directed acyclic graphs designed to represent the joint distribution of a set of variables. The circuits comprise three types of nodes: (1) leaf nodes, which correspond to input variables; (2) sum nodes, which compute weighted sums of their child nodes; and (3) product nodes, which compute products of their child nodes. When satisfying the properties of decomposability and smoothness, a probabilistic circuit becomes a tractable probabilistic model, ensuring efficient inferences over various distributions (Poon & Domingos, 2011). Specifically, joint, marginal, and conditional probabilities of input variables can be computed in at most two passes (from leaf nodes to the root node), with computational complexity linear in the size of the circuit. Consequently, probabilistic circuits combine the expressiveness of traditional graphical models with the scalability of modern deep learning frameworks.

Structure learning for probabilistic circuits aims to design structures that effectively balance expressiveness and computational efficiency.  Xia et al. (2023) categorize existing structure learning methods into four types: (1) handcrafted structure learning, where structures are manually designed for specific datasets (Gens & Domingos, 2012; Poon & Domingos, 2011; (2) data-based structure learning, which uses heuristic (Adel et al., 2015; Dennis & Ventura, 2012; Gens & Domingos, 2013; Krakovna & Looks, 2016; Molina et al., 2018; Rahman & Gogate, 2016; Rooshenas & Lowd, 2014; Vergari et al., 2015) or non-heuristic algorithms (Lee et al., 2014; Peharz et al., 2014, 2019; Trapp et al., 2016) to learn structures from data; (3) random structure learning, where structures are randomly generated as a flexible starting point (Peharz et al., 2019; Rashwan et al., 2016; Trapp et al., 2019); and (4) ensemble structure learning, which combines multiple structures to improve generalization to high-dimensional data (Ventola et al., 2020). In this paper, we utilize the first and second types of structure learning approaches to embed explicit and implicit logical rules, respectively.

Parameter learning for probabilistic circuits involves finding optimal parameters for a given structure, enabling the circuits to accurately capture the underlying probability distributions within the observed data. Parameter learning can be broadly categorized into two types: generative and discriminative. Generative parameter learning (Peharz, 2015; Poon & Domingos, 2011; Rashwan et al., 2016; Zhao et al., 2016, 2016), the most common paradigm, aims to maximize the joint probabilities of all variables. The generative approach is particularly suited for tasks such as density estimation, generative modeling, and probabilistic reasoning. In contrast, discriminative parameter learning (Adel et al., 2015; Gens & Domingos, 2012; Rashwan et al., 2018) focuses on maximizing the conditional probabilities of a class variable given other variables, making it ideal for classification and regression tasks. In this paper, we adopt CCCP (Zhao et al., 2016), a generative parameter learning approach, as it admits multiplicative parameter updates that provide a monotonic increase in the log-likelihood and lead to faster and more stable convergence.

On the other hand, some works develop probabilistic circuits parameterized by neural networks. For instance, Ahmed et al. (2022) use an amortized neural network to output the weights in circuits. To address the overfitting problem of large probabilistic circuits, Shih et al. (2021) exploit the generalization ability of a small-scale neural network and employ it to generate the parameters of a large circuit. Shao et al. (2022) aims to learn a probabilistic circuit to model the conditional distribution of target variables given input variables. In their approach, a neural network, conditioned on the input variables, is utilized to generate the circuit’s parameters. This integration of neural networks enhances the expressiveness of probabilistic circuits and has been used in neuro-symbolic integration (Ahmed et al., 2022; Manhaeve et al., 2018). In this paper, the circuit’s integration with the attribute recognition model can be seen as parameterizing the input distributions of the circuit, instead of the weights of the circuit. The final predictions are a result of utilizing the outputs of a circuit through the law of total probability.

***Integration of Probabilistic Graphical Models***

Probabilistic graphical models (PGMs) are frameworks that use graphs to express conditional dependencies between variables and represent their joint probability distributions. With their expressiveness, PGMs can be integrated into the decision-making process to enhance models from various perspectives. Our work demonstrates one approach to integrating PGMs, i.e., probabilistic circuits, to improve the transparency and interpretability of a model’s predictive process. In contrast, Yang et al. (2022), Gürel et al. (2021), Zhang et al. (2023), and Kang et al. (2024) have focused on leveraging PGMs integration to enhance the adversarial robustness of deep classification models.

***Neuro-Symbolic Learning***

Neuro-symbolic learning integrates neural networks with symbolic representations, combining data-driven learning with symbolic reasoning to leverage the strengths of both. Variants of CBMs that embed inherent rules within the task predictor serve as one exemplifying application of neuro-symbolic learning. Beyond CBMs, this neuro-symbolic paradigm can be implemented in various other forms. One line of research focuses on designing symbolic-based objective functions. For instance, Badreddine et al. (2022) propose objectives that maximize the satisfiability of predefined symbolic rules over a neural network’s outputs. Similarly, Xu et al. (2018) and Ahmed et al. (2023) define objectives that maximize the probabilities of generating outputs aligned with symbolic rules. These objectives can also function as regularization terms alongside standard classification losses, encouraging a neural network to adhere to specific rules by optimizing its parameters accordingly. Another line of research emphasizes the design of model architectures. For example, Ahmed et al. (2022) introduce a semantic probabilistic layer, a predictive layer designed for structured-output predictions, which can be seamlessly integrated into neural networks to ensure predictions align with certain symbolic constraints. Overall, these works ensure that learned models follow specific symbolic rules, either through the design of objective functions or the modification of model architectures. However, while these approaches enforce rule compliance, the explicit meaning of individual model components often remains unclear, raising concerns about their transparency and interoperability.

***Knowledge Compilation***

In the field of knowledge compilation (Darwiche & Marquis, 2002), previous studies have made efforts to compile PGMs (Chavira & Darwiche, 2005; Choi et al., 2013) or logical formulas (Chavira & Darwiche, 2005; Darwiche, 2002; Pipatsrisawat & Darwiche, 2008) into computationally efficient structures that support various inference tasks. In this paper, the proposed knowledge-injected parameter learning approach adopts a simple compilation strategy that compiles a set of weighted AND rules into a two-layer probabilistic circuit. Specifically, each product node corresponds to an individual AND rule, while the sum node encodes the weights associated with these rules.

## Conclusions

In this paper, we propose Neural Probabilistic Circuits (NPCs), a novel architecture that decomposes the decision-making process into attribute recognition and logical reasoning, enabling compositional and interpretable predictions. Experimental results across four image classification datasets demonstrate that NPC delivers competitive performance when compared with four baseline models. In addition, we conduct a series of ablation studies and observe the following findings: (1) Compared with individual binary concepts, utilizing attributes preserves relational constraints, thus enhancing model performance. (2) Insufficient attributes compromise NPC’s performance on downstream tasks, and the impact of excluding specific attributes varies depending on their inherent properties. (3) For simple datasets, manually constructed circuits using the knowledge-injected approach are sufficient to result in competitive performance, whereas data-driven circuits are more effective on complex datasets. (4) Joint optimization leads to further improvements in the model performance. Furthermore, we demonstrate how the provided most probable explanations and counterfactual explanations offer attribute-level insights into the model’s decision-making process, thereby enhancing the human’s understanding. Finally, we discuss the limitations and potential future research directions for NPCs from various perspectives, providing insights for future improvements. Our work demonstrates the potential of NPCs to enhance model interpretability and performance by integrating semantically meaningful attributes with probabilistic circuits, offering actionable insights for future advancements in interpretable machine learning through logical reasoning.

## Data Availability

We employed four publicly available image datasets in this work, namely, Animal with Attributes 2 (AwA2), CelebFaces Attributes (CelebA), German Traffic Sign Recognition Benchmark (GTSRB), and Modified National Institute of Standards and Technology (MNIST). AwA2 is available at https://cvml.ista.ac.at/AwA2/. CelebA is available at https://mmlab.ie.cuhk.edu.hk/projects/CelebA.html. GTSRB is availble at https://www.kaggle.com/datasets/meowmeowmeowmeowmeow/gtsrb-german-traffic-sign. MNIST is available at https://huggingface.co/datasets/ylecun/mnist. Three out of the four datasets, namely, AwA2, GTSRB, and MNIST, grants us rights to freely use, redistribute, and publish portions of the datasets for research purposes. CelebA, albeit granting limited access for non-commercial research purposes, explicitly forbids redistributions or publications of any portion of the dataset, as per its terms of use. Therefore, all instances of CelebA images are redacted from this work in compliance with such terms of use. During our experiments, we have generated additional data for all four datasets, such as annotations in regards to attribute recognition. Upon acceptance, all data generated during our experiments will be released and remain publicly available under appropriate licenses.

## Appendix 1: Joint Optimization

In this section, we provide additional algorithmic details for the proposed joint optimization approach. The approach aims to jointly optimize the attribute recognition model and the circuit-based task predictor in an NPC using the loss function defined in Eq. (3). Specifically, we employ the stochastic gradient descent algorithm to optimize the parameters of the attribute recognition model, i.e., *θ *, and use the projected gradient descent algorithm to optimize the parameters of the circuit, i.e., *w*, to ensure their positivity.

***Optimizing*** *θ *

Let $\eta _{A}$ denote the learning rate for the attribute recognition model. The parameters of the model at the *(t+1)*th optimization step are as follows,

$$\begin{aligned} \theta ^{(t+1)}&= \theta ^{(t)}+\eta _{A} \cdot \sum _{(x, y)\in D}\frac{\partial \log {\operatorname {Pr}}_{\theta ^{(t)}, w^{(t)}} (Y=y \mid X=x)}{\partial \theta }.\end{aligned} $$

Here, ${\operatorname {Pr}}_{\theta ^{(t)}, w^{(t)}}(Y=y \mid X=x)$ is computed using Eq. (1).

***Optimizing*** *w*

Let $\eta _{C}$ denote the learning rate for the circuit. The *d*th weight at the *(t+1)*th optimization step is as follows,

$$\begin{aligned} w_{d}^{(t+1)}&= {\mathcal {P}}_{\textbf{R}_{++}}\left\{ w_{d}^{(t)}+\eta _{C} \cdot \sum _{(x, y)\in D}\frac{\partial \log {\operatorname {Pr}}_{\theta ^{(t)}, w^{(t)}} (Y=y \mid X=x)}{\partial w_{d}}\right\} \\&= {\mathcal {P}}_{\textbf{R}_{++}}\left\{ w_{d}^{(t)}+\eta _{C} \cdot \sum _{(x, y)\in D}\frac{1}{{\operatorname {Pr}}_{\theta ^{(t)}, w^{(t)}} (Y=y \mid X=x)}\frac{\partial {\operatorname {Pr}}_{\theta ^{(t)}, w^{(t)}} (Y=y \mid X=x)}{\partial w_{d}}\right\} . \end{aligned}$$

Here, $\Pr _{\theta ^{(t)}, w^{(t)}} (Y=y \mid X=x)$ is computed using Eq. (1), and the gradient term is given by,

$$\begin{aligned}&\frac{\partial {\operatorname {Pr}}_{\theta ^{(t)}, w^{(t)}}\left( Y=y \mid X=x\right) }{\partial w_{d}} = \sum _{a_{1:K}} \prod _{k=1}^{K} {\operatorname {Pr}}_{\theta ^{(t)}}\left( A_{k}=a_{k} \mid X=x\right) \cdot \frac{\partial {\operatorname {Pr}}_{w^{(t)}}\left( Y=y \mid A_{1:K}=a_{1:K}\right) }{\partial w_{d}} \\&\quad = \sum _{a_{1:K}} \prod _{k=1}^{K} {\operatorname {Pr}}_{\theta ^{(t)}}\left( A_{k}=a_{k} \mid X=x\right) \cdot \frac{f_{S}\left( y, a_{1:K}; w^{(t)}\right) }{f_{S}\left( \emptyset , a_{1:K}; w^{(t)}\right) } \cdot \left[ \frac{\partial \log f_{S}\left( y, a_{1:K}; w^{(t)}\right) }{\partial w_{d}}-\frac{\partial \log f_{S}\left( \emptyset , a_{1:K}; w^{(t)}\right) }{\partial w_{d}} \right] . \end{aligned}$$

In particular, $\prod _{k=1}^{K} {\operatorname {Pr}}_{\theta ^{(t)}}\left( A_{k}=a_{k} \mid X=x\right)$ and $\frac{f_{S}\left( y, a_{1:K}; w^{(t)}\right) }{f_{S}\left( \emptyset , a_{1:K}; w^{(t)}\right) }$ are provided by the attribute recognition model and the circuit, respectively. $\frac{\partial \log f_{S}\left( y, a_{1:K}; w^{(t)}\right) }{\partial w_{d}}$ and $\frac{\partial \log f_{S}\left( \emptyset , a_{1:K}; w^{(t)}\right) }{\partial w_{d}}$ can be computed recursively within a circuit (Poon & Domingos, 2011).

## Appendix 2: Proof for Compositional Error

In this section, we present a detailed proof for Theorem 2. Throughout the proof, expressions with parameters such as $\Pr _{\theta}$, $\Pr _{w}$, and $\Pr _{\theta , w}$ refer to probabilities learned by the models, while those without parameters represent ground-truth probabilities.

5 $$\begin{aligned} \epsilon _{\theta , w}&= {\mathbb {E}}_{X}\left[ \frac{1}{2}\sum _{y} \left| \Pr _{\theta , w}(Y=y\mid X) - \Pr (Y=y\mid X)\right| \right] \\&\leqslant {\mathbb {E}}_{X}\bigg [ \frac{1}{2}\sum _{y} \sum _{a_{1:K}}\bigg | \prod _{k}\Pr _{\theta} (A_{k}=a_{k}\mid X)\cdot \Pr _{w}(Y=y\mid A_{1:K}=a_{1:K}) - \prod _{k}\Pr (A_{k}=a_{k}\mid X)\cdot \Pr _{w}(Y=y\mid A_{1:K}=a_{1:K}) \\&\phantom {\leqslant {\mathbb {E}}_{X}[ \frac{1}{2}\sum _{y} \sum _{a_{1:K}}} + \prod _{k}\Pr (A_{k}=a_{k}\mid X)\cdot \Pr _{w}(Y=y\mid A_{1:K}=a_{1:K}) - \prod _{k}\Pr (A_{k}=a_{k}\mid X)\cdot \Pr (Y=y\mid A_{1:K}=a_{1:K}) \bigg | \bigg ] \\&\leqslant {\mathbb {E}}_{X}\left[ \frac{1}{2}\sum _{y}\sum _{a_{1:K}} \left| \prod _{k}\Pr _{\theta} (A_{k}=a_{k}\mid X)-\prod _{k}\Pr (A_{k}=a_{k}\mid X) \right| \cdot \Pr _{w}(Y=y\mid A_{1:K}=a_{1:K}) \right] \end{aligned}$$

6 $$\begin{aligned}&\phantom {\quad } + {\mathbb {E}}_{X}\left[ \frac{1}{2}\sum _{y}\sum _{a_{1:K}} \prod _{k}\Pr (A_{k}=a_{k}\mid X)\cdot \left| \Pr _{w}(Y=y\mid A_{1:K}=a_{1:K})-\Pr (Y=y\mid A_{1:K}=a_{1:K}) \right| \right] . \end{aligned}$$

Thus, the upper bound of $\epsilon _{\theta , w}$ is decomposed into two terms.

We derive the upper bound for the first term, i.e., Eq. (5), as follows,

$$\begin{aligned} {\text {Eq. (5)}}&= {\mathbb {E}}_{X}\left[ \frac{1}{2}\sum _{a_{1:K}} \left| \prod _{k}\Pr _{\theta} (A_{k}=a_{k}\mid X)-\prod _{k}\Pr (A_{k}=a_{k}\mid X) \right| \right] = {\mathbb {E}}_{X}\left[ d_{\text {TV}}\left( \Pr _{\theta} (A_{1:K}\mid X), \Pr (A_{1:K}\mid X) \right) \right] = \epsilon _{\theta} \\&\leqslant {\mathbb {E}}_{X}\left[ \frac{1}{2}\sum _{k}\sum _{a_{k}}\left| \Pr _{\theta} (A_{k}=a_{k}\mid X)-\Pr (A_{k}=a_{k}\mid X) \right| \right] = \sum _{k}{\mathbb {E}}_{X}\left[ d_{\text {TV}}\left( \Pr _{\theta} (A_{k}=a_{k}\mid X),\Pr (A_{k}=a_{k}\mid X) \right) \right] = \sum _{k}\epsilon _{\theta} ^{k}. \end{aligned}$$

We derive the upper bound for the second term, i.e., Eq. (6), as follows,

$$\begin{aligned} {\text {Eq. (6)}}&= \frac{1}{2}\sum _x\sum _{y}\sum _{a_{1:K}}\Pr (X=x, A_{1:K}=a_{1:K})\cdot \left| \Pr _{w}(Y=y\mid A_{1:K}=a_{1:K}) - \Pr (Y=y\mid A_{1:K}=a_{1:K}) \right| \\&= \frac{1}{2}\sum _{y}\sum _{a_{1:K}}\Pr (A_{1:K}=a_{1:K})\cdot \left| \Pr _{w}(Y=y\mid A_{1:K}=a_{1:K}) - \Pr (Y=y\mid A_{1:K}=a_{1:K}) \right| \\&= \frac{1}{2}\sum _{y}\sum _{a_{1:K}} | \Pr (A_{1:K}=a_{1:K})\cdot \Pr _{w}(Y=y\mid A_{1:K}=a_{1:K}) - \Pr _{w}(A_{1:K}=a_{1:K})\cdot \Pr _{w}(Y=y\mid A_{1:K}=a_{1:K}) \\&\phantom {\frac{1}{2}\sum _{y}\sum _{a_{1:K}}\quad } + \Pr _{w}(A_{1:K}=a_{1:K})\cdot \Pr _{w}(Y=y\mid A_{1:K}=a_{1:K}) - \Pr (A_{1:K}=a_{1:K})\cdot \Pr (Y=y\mid A_{1:K}=a_{1:K})| \\&\leqslant \frac{1}{2}\sum _{y}\sum _{a_{1:K}} \Pr _{w}(Y=y\mid A_{1:K}=a_{1:K})\cdot \left| \Pr (A_{1:K}=a_{1:K})-\Pr _{w}(A_{1:K}=a_{1:K}) \right| \\&\phantom {\quad } +\frac{1}{2}\sum _{y}\sum _{a_{1:K}} \left| \Pr _{w}(Y=y, A_{1:K}=a_{1:K})-\Pr (Y=y, A_{1:K}=a_{1:K}) \right| \\&= \frac{1}{2}\sum _{a_{1:K}} \left| \Pr (A_{1:K}=a_{1:K})-\Pr _{w}(A_{1:K}=a_{1:K}) \right| + d_{\text {TV}}\left( \Pr _{w}(Y, A_{1:K}), \Pr (Y, A_{1:K}) \right) \\&= \frac{1}{2}\sum _{a_{1:K}} \left| \sum _{y}\Pr (Y=y, A_{1:K}=a_{1:K})-\sum _{y}\Pr _{w}(Y=y, A_{1:K}=a_{1:K}) \right| + d_{\text {TV}}\left( \Pr _{w}(Y, A_{1:K}), \Pr (Y, A_{1:K}) \right) \\&\leqslant \frac{1}{2}\sum _{a_{1:K}}\sum _{y} \left| \Pr (Y=y, A_{1:K}=a_{1:K})-\Pr _{w}(Y=y, A_{1:K}=a_{1:K}) \right| + d_{\text {TV}}\left( \Pr _{w}(Y, A_{1:K}), \Pr (Y, A_{1:K}) \right) \\&= 2d_{\text {TV}}\left( \Pr _{w}(Y, A_{1:K}), \Pr (Y, A_{1:K}) \right) = 2\epsilon _{w}. \end{aligned}$$

Combining results from Eqs. (5) and (6), we have $\epsilon _{\theta , w} \leqslant \epsilon _{\theta} + 2\epsilon _{w} \leqslant \sum _{k} \epsilon _{\theta} ^{k} + 2\epsilon _{w}$.

## Appendix 3: Experimental Setup

In this section, we provide additional details regarding the experimental setup.

***Model Architectures***

To ensure a fair comparison with the various baselines, we consistently adopt ResNet-34 (He et al., 2016) as the backbone for all methods. Specifically, in CBM (Koh et al., 2020), the concept recognition model is implemented using ResNet-34, where the output dimension of its final fully connected layer is modified to match the number of concepts. The task predictor is a linear layer that maps the predicted concept probabilities to the final class prediction scores. The architecture of Hybrid CBM (Mahinpei et al., 2021) is similar to that of CBM, except that the output dimension of the final fully connected layer in ResNet-34 is set to the sum of the number of concepts and the number of unsupervised neurons. In CEM (Zarlenga et al., 2022), the first module consists of two components: a ResNet-34, whose final fully connected layer output dimension is modified to n_hidden, and the proposed concept embedding layer. This embedding layer generates both concept embeddings and concept probabilities. The subsequent task predictor takes only the concept embeddings as input to produce the final class prediction scores. In DCR (Barbiero et al., 2023), the first module is identical to that of CEM, while the task predictor is the proposed deep concept reasoner. This reasoner takes concept probabilities as input and outputs class prediction scores, during which it leverages concept embeddings to formulate logical rules. In NPC, the attribute recognition model is implemented using a multi-task learning framework. Specifically, ResNet-34, without its final fully connected layer, serves as the feature extractor to capture common features across multiple attributes. For each attribute, a series of two linear layers acts as a dedicated task head, outputting a probability vector corresponding to the attribute. The task predictor in NPC is implemented as a probabilistic circuit, with detailed construction provided in Sect. 3.2.2.

***Training Details***

For most baseline methods, training is performed with a batch size of 256 over 150 epochs. For DCR, following Barbiero et al. (2023), the concept recognition model from CEM is used and kept frozen, and its task predictor is trained with a batch size of 256 for 3000 epochs. We use the SGD optimizer, configured with a learning rate of 0.01, a momentum of 0.9, and a weight decay of 0.00004. During training, we reduce the learning rate by a factor of 0.1 if the validation loss plateaus for 10 epochs. Additionally, the concept loss weight is set to 1 for CBM, CC-Hybrid-CBM, CEM, and DCR. The concept embedding size is configured to 16 for both CEM and DCR (Zarlenga et al., 2022), and in CEM, n_hidden is set equal to the concept embedding size. For Hybrid CBM, the number of unsupervised dimensions is set to the product of the number of concepts and (concept embedding size *- 1*). For NPC, the training process consists of three stages. In the first stage, the attribute recognition model is trained with a batch size of 256 over 150 epochs using the SGD optimizer. During training, we reduce the learning rate by a factor of 0.1 if the validation loss plateaus for 10 epochs. In the second stage, the circuit is learned and optimized following the procedures described by Gens and Domingos (2013) and Zhao et al. (2016), respectively. In the final stage, a learning rate of 0.01 is applied to the attribute recognition model while the circuit remains frozen, which is empirically shown to yield faster training and improved task performance. We run NPCs and all baseline models over five random seeds: 42, 52, 62, 72, and 82.

***Counterfactual Explanations***

We adopt Algorithm 1 to generate CEs over 100 iterations. The learning rate *γ * is set to 0.005 for MNIST-Addition, 0.01 for GTSRB, 0.1 for CelebA, and 0.05 for AwA2.

## Appendix 4: Attribute Configurations

Table 5 provides an overview of the attributes used, along with their corresponding values, for the four benchmark datasets. In MNIST-Addition and GTSRB, attributes are *single-valued*, meaning each sample is associated with only one value per attribute. In contrast, CelebA and AwA2 feature *multi-valued* attributes, allowing a sample to be associated with multiple values for a single attribute. For example, an image from the “zebra” class has both “black” and “white” as color attributes. Furthermore, attribute annotations in GTSRB, CelebA, and AwA2 are *class-wise*, where each class of samples all corresponds to the same specific set of attribute values. Conversely, in MNIST-Addition, samples from the same class may have different combinations of attribute values. For instance, a sample from the class “8” may correspond to attribute values such as “0, 8”, “1, 7”, or “8, 0”. Table 6 presents sample images from the four datasets.

**Table 5:** Attributes and their corresponding values for the four benchmark datasets

Dataset	Attribute	Values
MNIST-Addition	Number-First	0, 1, 2, 3, 4, 5, 6, 7, 8, 9
	Number-Second	0, 1, 2, 3, 4, 5, 6, 7, 8, 9
GTSRB	Color	Blue, Red, White
	Shape	Circle, Diamond, Octagon, Triangle
	Symbol	Arrow-Consecutive-Turns, Arrow-Down-Left, Arrow-Down-Right, Arrow-Left, Arrow-Right, Arrow-Roundabout, Arrow-Up, Arrow-Up-and-Left, Arrow-Up-and-Right, Bicycle, Bump, Car, Car-Truck, Car-Two, Deer, Exclamation-Mark, Ice-or-Snow, Person, Person-Two, Road-Narrows, Roadworks, Slash, Text, Traffic-Signal, Truck, Undefined
	Text	100, 120, 20, 30, 50, 60, 70, 80, Stop, Undefined
CelebA	Mouth	Mouth-Slightly-Open, None, Smiling
	Face	High-Cheekbones, None, Oval-Face
	Cosmetic	Heavy-Makeup, None, Wearing-Lipstick
	Hair	None, Wavy-Hair
	Appearance	Attractive, None
AwA2	Color	Black, Blue, Brown, Gray, None, Orange, Red, White, Yellow
	Surface	Furry, Hairless, Patches, Spots, Stripes, Toughskin
	Body	Bipedal, Bulbous, Horns, Lean, Longleg, Longneck, Quadrupedal, Tail, Tusks
	Limb	Claws, Flippers, Hands, Hooves, None, Pads, Paws

**Table 6:** Example images and their annotations from the four benchmark datasets

Image	Attribute	Values
	Number-First	3
	Number-Second	5
	Color	Red
	Shape	Octagon
	Symbol	Text
	Text	Stop
	Mouth	Mouth-Slightly-Open, Smiling
	Face	High-Cheekbones
	Cosmetic	Heavy-Makeup, Wearing-Lipstick
	Hair	Wavy-Hair
	Appearance	Attractive
	Color	Black, White
	Surface	Furry, Stripes, Toughskin
	Body	Lean, Longleg, Longneck, Quadrupedal, Tail
	Limb	Hooves

The CelebA image is redacted in compliance with its terms of use

## Appendix 5: Human-Predefined Logical Rules

Human experience can be used to formulate specific logical rules for downstream tasks, representing valuable domain knowledge that can be integrated into models to enhance their reliability. Here, we demonstrate a two-step procedure for constructing logical rules based on a given set of observed samples ${\bar{D}}=\{(x, y, a_{1:K})\}$. (1) For each observed sample $(x, y, a_{1:K})$, we establish a corresponding logical rule of the form ${\mathbb {I}}(A_{1}=a_{1}) \wedge \cdots \wedge {\mathbb {I}}(A_{K}=a_{K}) \wedge {\mathbb {I}}(Y=y)$. (2) The occurrences of the logical rules derived from all observed samples are then normalized to form a weight for each rule, ensuring that the rules reflect the distribution of the observed data. Tables 7 and 8 illustrates the rules constructed using training samples from the MNIST-Addition and GTSRB datasets. In addition to the standardized procedure introduced above, humans can also leverage their expertise to incorporate more diverse and task-specific rules beyond this form

**Table 7:** Logical rules constructed using training samples from MNIST-Addition and GTSRB datasets (Part 1)

Dataset	Logical rules
MNIST-Addition	$$\begin{aligned}&{\mathbb {I}}(\text {Number-First}=a_{1}) \wedge {\mathbb {I}}(\text {Number-Second}=a_{2})\\ &\wedge {\mathbb {I}}({\text {Class}}=a_{1}+a_{2}),~a_{1},a_{2}\in [0,9]\end{aligned}$$
GTSRB	$$\begin{aligned}&{\mathbb {I}}({\text {Color}}={\text {Red}}) \wedge {\mathbb {I}}({\text {Shape}}={\text {Circle}}) \wedge {\mathbb {I}}({\text {Symbol}}={\text {Text}})\\ & \wedge {\mathbb {I}}({\text {Text}}=\text {20}) \wedge {\mathbb {I}}({\text {Class}}={\text {regulatory}}{-}{\text {maximum-speed-limit-20}})\end{aligned}$$
	$$\begin{aligned}&{\mathbb {I}}({\text {Color}}={\text {Red}}) \wedge {\mathbb {I}}({\text {Shape}}={\text {Circle}}) \wedge {\mathbb {I}}({\text {Symbol}}={\text {Text}}) \\ & \wedge {\mathbb {I}}({\text {Text}}=\text {30}) \wedge {\mathbb {I}}({\text {Class}}={\text {regulatory}}{-}{\text {maximum-speed-limit-30}})\end{aligned}$$
	$$\begin{aligned}&{\mathbb {I}}({\text {Color}}={\text {Red}}) \wedge {\mathbb {I}}({\text {Shape}}={\text {Circle}}) \wedge {\mathbb {I}}({\text {Symbol}}={\text {Text}})\\& \wedge {\mathbb {I}}({\text {Text}}=\text {50}) \wedge {\mathbb {I}}({\text {Class}}={\text {regulatory}}{-}{\text {maximum-speed-limit-50}})\end{aligned}$$
	$$\begin{aligned}&{\mathbb {I}}({\text {Color}}={\text {Red}}) \wedge {\mathbb {I}}({\text {Shape}}={\text {Circle}}) \wedge {\mathbb {I}}({\text {Symbol}}={\text {Text}}) \\&\wedge {\mathbb {I}}({\text {Text}}=\text {60}) \wedge {\mathbb {I}}({\text {Class}}={\text {regulatory}}{-}{\text {maximum-speed-limit-60}})\end{aligned}$$
	$$\begin{aligned}&{\mathbb {I}}({\text {Color}}={\text {Red}}) \wedge {\mathbb {I}}({\text {Shape}}={\text {Circle}}) \wedge {\mathbb {I}}({\text {Symbol}}={\text {Text}}) \\&\wedge {\mathbb {I}}({\text {Text}}=\text {70}) \wedge {\mathbb {I}}({\text {Class}}={\text {regulatory}}{-}{\text {maximum-speed-limit-70}})\end{aligned}$$
	$$\begin{aligned}&{\mathbb {I}}({\text {Color}}={\text {Red}}) \wedge {\mathbb {I}}({\text {Shape}}={\text {Circle}}) \wedge {\mathbb {I}}({\text {Symbol}}={\text {Text}}) \wedge {\mathbb {I}}({\text {Text}}=\text {80}) \\ & \wedge {\mathbb {I}}({\text {Class}}={\text {regulatory}}{-}{\text {maximum-speed-limit-80}})\end{aligned}$$
	$$\begin{aligned}&{\mathbb {I}}({\text {Color}}={\text {Red}}) \wedge {\mathbb {I}}({\text {Shape}}={\text {Circle}}) \wedge {\mathbb {I}}({\text {Symbol}}={\text {Text}})\\& \wedge {\mathbb {I}}({\text {Text}}=\text {100}) \wedge {\mathbb {I}}({\text {Class}}={\text {regulatory}}{-}{\text {maximum-speed-limit-100}})\end{aligned}$$
	$$\begin{aligned}&{\mathbb {I}}({\text {Color}}={\text {Red}}) \wedge {\mathbb {I}}({\text {Shape}}={\text {Circle}}) \wedge {\mathbb {I}}({\text {Symbol}}={\text {Text}})\\& \wedge {\mathbb {I}}({\text {Text}}=\text {120}) \wedge {\mathbb {I}}({\text {Class}}={\text {regulatory}}{-}{\text {maximum-speed-limit-120}})\end{aligned}$$
	$$\begin{aligned}&{\mathbb {I}}({\text {Color}}={\text {White}}) \wedge {\mathbb {I}}({\text {Shape}}={\text {Circle}}) \wedge {\mathbb {I}}({\text {Symbol}}={\text {Text}})\\& \wedge {\mathbb {I}}({\text {Text}}=\text {80}) \wedge {\mathbb {I}}({\text {Class}}={\text {regulatory}}{-}{\text {end-of-maximum-speed-limit-80}})\end{aligned}$$
	$$\begin{aligned}&{\mathbb {I}}({\text {Color}}={\text {Red}}) \wedge {\mathbb {I}}({\text {Shape}}={\text {Circle}}) \wedge {\mathbb {I}}({\text {Symbol}}=\text {Cat-Two})\\& \wedge {\mathbb {I}}({\text {Text}}={\text {Undefined}}) \wedge {\mathbb {I}}({\text {Class}}={\text {regulatory}}{-}{\text {no-overtaking}})\end{aligned}$$
	$$\begin{aligned}&{\mathbb {I}}({\text {Color}}={\text {Red}}) \wedge {\mathbb {I}}({\text {Shape}}={\text {Circle}}) \wedge {\mathbb {I}}({\text {Symbol}}={\text {Car-Truck}})\\& \wedge {\mathbb {I}}({\text {Text}}={\text {Undefined}}) \\ & \wedge {\mathbb {I}}({\text {Class}}={\text {regulatory}}{-}{\text {no-overtaking-by-heavy-goods-vehicles}})\end{aligned}$$
	$$\begin{aligned}&{\mathbb {I}}({\text {Color}}={\text {Red}}) \wedge {\mathbb {I}}({\text {Shape}}={\text {Triangle}}) \wedge {\mathbb {I}}({\text {Symbol}}={\text {Arrow-Up}}) \\&\wedge {\mathbb {I}}({\text {Text}}={\text {Undefined}}) \wedge {\mathbb {I}}({\text {Class}}={\text {warning}}{-}{\text {crossroads}})\end{aligned}$$
	$$\begin{aligned}&{\mathbb {I}}({\text {Color}}={\text {White}}) \wedge {\mathbb {I}}({\text {Shape}}={\text {Diamond}}) \wedge {\mathbb {I}}({\text {Symbol}}={\text {Undefined}}) \\&\wedge {\mathbb {I}}({\text {Text}}={\text {Undefined}}) \wedge {\mathbb {I}}({\text {Class}}={\text {regulatory}}{-}{\text {priority-road}})\end{aligned}$$
	$$\begin{aligned}&{\mathbb {I}}({\text {Color}}={\text {Red}}) \wedge {\mathbb {I}}({\text {Shape}}={\text {Triangle}}) \wedge {\mathbb {I}}({\text {Symbol}}={\text {Undefined}})\\& \wedge {\mathbb {I}}({\text {Text}}={\text {Undefined}}) \wedge {\mathbb {I}}({\text {Class}}={\text {regulatory}}{-}{\text {yield}})\end{aligned}$$
	$$\begin{aligned}&{\mathbb {I}}({\text {Color}}={\text {Red}}) \wedge {\mathbb {I}}({\text {Shape}}={\text {Octagon}}) \wedge {\mathbb {I}}({\text {Symbol}}={\text {Text}}) \\&\wedge {\mathbb {I}}({\text {Text}}={\text {Stop}}) \wedge {\mathbb {I}}({\text {Class}}={\text {regulatory}}{-}{\text {stop}})\end{aligned}$$
	$$\begin{aligned}&{\mathbb {I}}({\text {Color}}={\text {Red}}) \wedge {\mathbb {I}}({\text {Shape}}={\text {Circle}}) \wedge {\mathbb {I}}({\text {Symbol}}={\text {Undefined}})\\& \wedge {\mathbb {I}}({\text {Text}}={\text {Undefined}}) \wedge {\mathbb {I}}({\text {Class}}={\text {regulatory}}{-}{\text {road-closed-to-vehicles}})\end{aligned}$$
	$$\begin{aligned}&{\mathbb {I}}({\text {Color}}={\text {Red}}) \wedge {\mathbb {I}}({\text {Shape}}={\text {Circle}}) \wedge {\mathbb {I}}({\text {Symbol}}=\text {Truck}) \\&\wedge {\mathbb {I}}({\text {Text}}={\text {Undefined}}) \wedge {\mathbb {I}}({\text {Class}}={\text {regulatory}}{-}{\text {no-heavy-goods-vehicles}})\end{aligned}$$
	$$\begin{aligned}&{\mathbb {I}}({\text {Color}}={\text {Red}}) \wedge {\mathbb {I}}({\text {Shape}}={\text {Circle}}) \wedge {\mathbb {I}}({\text {Symbol}}=\text {Slash})\\& \wedge {\mathbb {I}}({\text {Text}}={\text {Undefined}}) \wedge {\mathbb {I}}({\text {Class}}={\text {regulatory}}{-}{\text {no-entry}})\end{aligned}$$
	$$\begin{aligned}&{\mathbb {I}}({\text {Color}}={\text {Red}}) \wedge {\mathbb {I}}({\text {Shape}}={\text {Triangle}}) \wedge {\mathbb {I}}({\text {Symbol}}={\text {Exclamation-Mark}})\\& \wedge {\mathbb {I}}({\text {Text}}={\text {Undefined}}) \wedge {\mathbb {I}}({\text {Class}}={\text {warning}}{-}{\text {other-danger}})\end{aligned}$$
	$$\begin{aligned}&{\mathbb {I}}({\text {Color}}={\text {Red}}) \wedge {\mathbb {I}}({\text {Shape}}={\text {Triangle}}) \wedge {\mathbb {I}}({\text {Symbol}}={\text {Arrow-Left}})\\& \wedge {\mathbb {I}}({\text {Text}}={\text {Undefined}}) \wedge {\mathbb {I}}({\text {Class}}={\text {warning}}{-}{\text {curve-left}})\end{aligned}$$
	$$\begin{aligned}&{\mathbb {I}}({\text {Color}}={\text {Red}}) \wedge {\mathbb {I}}({\text {Shape}}={\text {Triangle}}) \wedge {\mathbb {I}}({\text {Symbol}}={\text {Arrow-Right}})\\& \wedge {\mathbb {I}}({\text {Text}}={\text {Undefined}}) \wedge {\mathbb {I}}({\text {Class}}={\text {warning}}{-}{\text {curve-right}})\end{aligned}$$
	$$\begin{aligned}&{\mathbb {I}}({\text {Color}}={\text {Red}}) \wedge {\mathbb {I}}({\text {Shape}}={\text {Triangle}}) \wedge {\mathbb {I}}({\text {Symbol}}={\text {Arrow-Consecutive-Turns}})\\& \wedge {\mathbb {I}}({\text {Text}}={\text {Undefined}}) \wedge {\mathbb {I}}({\text {Class}}={\text {warning}}{-}{\text {double-curve-first-left}})\end{aligned}$$
	$$\begin{aligned}&{\mathbb {I}}({\text {Color}}={\text {Red}}) \wedge {\mathbb {I}}({\text {Shape}}={\text {Triangle}}) \wedge {\mathbb {I}}({\text {Symbol}}={\text {Bump}})\\& \wedge {\mathbb {I}}({\text {Text}}={\text {Undefined}}) \wedge {\mathbb {I}}({\text {Class}}={\text {warning}}{-}{\text {uneven-road}})\end{aligned}$$
	$$\begin{aligned}&{\mathbb {I}}({\text {Color}}={\text {Red}}) \wedge {\mathbb {I}}({\text {Shape}}={\text {Triangle}}) \wedge {\mathbb {I}}({\text {Symbol}}={\text {Car}}) \\&\wedge {\mathbb {I}}({\text {Text}}={\text {Undefined}}) \wedge {\mathbb {I}}({\text {Class}}={\text {warning}}{-}{\text {slippery-road-surface}})\end{aligned}$$

**Table 8:** Logical rules constructed using training samples from MNIST-Addition and GTSRB datasets (Part 2)

Dataset	Logical rules
GTSRB	$$\begin{aligned}&{\mathbb {I}}({\text {Color}}={\text {Red}}) \wedge {\mathbb {I}}({\text {Shape}}={\text {Triangle}}) \wedge {\mathbb {I}}({\text {Symbol}}={\text {Road-Narrows}}) \\& \wedge {\mathbb {I}}({\text {Text}}={\text {Undefined}}) \wedge {\mathbb {I}}({\text {Class}}={\text {warning}}{-}{\text {road-narrows-right}})\end{aligned}$$
	$$\begin{aligned}&{\mathbb {I}}({\text {Color}}={\text {Red}}) \wedge {\mathbb {I}}({\text {Shape}}={\text {Triangle}}) \wedge {\mathbb {I}}({\text {Symbol}}={\text {Roadworks}}) \\& \wedge {\mathbb {I}}({\text {Text}}={\text {Undefined}}) \wedge {\mathbb {I}}({\text {Class}}={\text {warning}}{-}{\text {roadworks}})\end{aligned}$$
	$$\begin{aligned}&{\mathbb {I}}({\text {Color}}={\text {Red}}) \wedge {\mathbb {I}}({\text {Shape}}={\text {Triangle}}) \wedge {\mathbb {I}}({\text {Symbol}}={\text {Traffic-Signal}}) \\& \wedge {\mathbb {I}}({\text {Text}}={\text {Undefined}}) \wedge {\mathbb {I}}({\text {Class}}={\text {warning}}{-}{\text {traffic-signals}})\end{aligned}$$
	$$\begin{aligned}&{\mathbb {I}}({\text {Color}}={\text {Red}}) \wedge {\mathbb {I}}({\text {Shape}}={\text {Triangle}}) \wedge {\mathbb {I}}({\text {Symbol}}={\text {Person}})\\& \wedge {\mathbb {I}}({\text {Text}}={\text {Undefined}}) \wedge {\mathbb {I}}({\text {Class}}={\text {warning}}{-}{\text {pedestrians-crossing}})\end{aligned}$$
	$$\begin{aligned}&{\mathbb {I}}({\text {Color}}={\text {Red}}) \wedge {\mathbb {I}}({\text {Shape}}={\text {Triangle}}) \wedge {\mathbb {I}}({\text {Symbol}}={\text {Person-Two}}) \\& \wedge {\mathbb {I}}({\text {Text}}={\text {Undefined}}) \wedge {\mathbb {I}}({\text {Class}}={\text {warning}}{-}{\text {children}})\end{aligned}$$
	$$\begin{aligned}&{\mathbb {I}}({\text {Color}}={\text {Red}}) \wedge {\mathbb {I}}({\text {Shape}}={\text {Triangle}}) \wedge {\mathbb {I}}({\text {Symbol}}={\text {Bicycle}}) \\& \wedge {\mathbb {I}}({\text {Text}}={\text {Undefined}}) \wedge {\mathbb {I}}({\text {Class}}={\text {warning}}{-}{\text {children}})\end{aligned}$$
	$$\begin{aligned}&{\mathbb {I}}({\text {Color}}={\text {Red}}) \wedge {\mathbb {I}}({\text {Shape}}={\text {Triangle}}) \wedge {\mathbb {I}}({\text {Symbol}}={\text {Ice-or-Snow}}) \\& \wedge {\mathbb {I}}({\text {Text}}={\text {Undefined}}) \wedge {\mathbb {I}}({\text {Class}}={\text {warning}}{-}{\text {ice-or-snow}})\end{aligned}$$
	$$\begin{aligned}&{\mathbb {I}}({\text {Color}}={\text {Red}}) \wedge {\mathbb {I}}({\text {Shape}}={\text {Triangle}}) \wedge {\mathbb {I}}({\text {Symbol}}={\text {Deer}})\\& \wedge {\mathbb {I}}({\text {Text}}={\text {Undefined}}) \wedge {\mathbb {I}}({\text {Class}}={\text {warning}}{-}{\text {wild-animals}})\end{aligned}$$
	$$\begin{aligned}&{\mathbb {I}}({\text {Color}}={\text {White}}) \wedge {\mathbb {I}}({\text {Shape}}={\text {Circle}}) \wedge {\mathbb {I}}({\text {Symbol}}={\text {Undefined}})\\& \wedge {\mathbb {I}}({\text {Text}}={\text {Undefined}}) \wedge {\mathbb {I}}({\text {Class}}={\text {regulatory}}{-}{\text {end-of-prohibition}})\end{aligned}$$
	$$\begin{aligned}&{\mathbb {I}}({\text {Color}}={\text {Blue}}) \wedge {\mathbb {I}}({\text {Shape}}={\text {Circle}}) \wedge {\mathbb {I}}({\text {Symbol}}={\text {Arrow-Right}})\\& \wedge {\mathbb {I}}({\text {Text}}={\text {Undefined}}) \wedge {\mathbb {I}}({\text {Class}}={\text {regulatory}}{-}{\text {turn-right-ahead}})\end{aligned}$$
	$$\begin{aligned}&{\mathbb {I}}({\text {Color}}={\text {Blue}}) \wedge {\mathbb {I}}({\text {Shape}}={\text {Circle}}) \wedge {\mathbb {I}}({\text {Symbol}}={\text {Arrow-Left}})\\& \wedge {\mathbb {I}}({\text {Text}}={\text {Undefined}}) \wedge {\mathbb {I}}({\text {Class}}={\text {regulatory}}{-}{\text {turn-left-ahead}})\end{aligned}$$
	$$\begin{aligned}&{\mathbb {I}}({\text {Color}}={\text {Blue}}) \wedge {\mathbb {I}}({\text {Shape}}={\text {Circle}}) \wedge {\mathbb {I}}({\text {Symbol}}={\text {Arrow-Up}})\\& \wedge {\mathbb {I}}({\text {Text}}={\text {Undefined}}) \wedge {\mathbb {I}}({\text {Class}}={\text {regulatory}}{-}{\text {go-straight}})\end{aligned}$$
	$$\begin{aligned}&{\mathbb {I}}({\text {Color}}={\text {Blue}}) \wedge {\mathbb {I}}({\text {Shape}}={\text {Circle}}) \wedge {\mathbb {I}}({\text {Symbol}}={\text {Arrow-Up and Right}}) \\& \wedge {\mathbb {I}}({\text {Text}}={\text {Undefined}}) \wedge {\mathbb {I}}({\text {Class}}={\text {regulatory}}{-}{\text {go-straight-or-right}})\end{aligned}$$
	$$\begin{aligned}&{\mathbb {I}}({\text {Color}}={\text {Blue}}) \wedge {\mathbb {I}}({\text {Shape}}={\text {Circle}}) \wedge {\mathbb {I}}({\text {Symbol}}={\text {Arrow-Up and Left}}) \\& \wedge {\mathbb {I}}({\text {Text}}={\text {Undefined}}) \wedge {\mathbb {I}}({\text {Class}}={\text {regulatory}}{-}{\text {go-straight-or-left}})\end{aligned}$$
	$$\begin{aligned}&{\mathbb {I}}({\text {Color}}={\text {Blue}}) \wedge {\mathbb {I}}({\text {Shape}}={\text {Circle}}) \wedge {\mathbb {I}}({\text {Symbol}}={\text {Arrow-Down-Right}}) \\& \wedge {\mathbb {I}}({\text {Text}}={\text {Undefined}}) \wedge {\mathbb {I}}({\text {Class}}={\text {regulatory}}{-}{\text {keep-right}})\end{aligned}$$
	$$\begin{aligned}&{\mathbb {I}}({\text {Color}}={\text {Blue}}) \wedge {\mathbb {I}}({\text {Shape}}={\text {Circle}}) \wedge {\mathbb {I}}({\text {Symbol}}={\text {Arrow-Down-Left}}) \\& \wedge {\mathbb {I}}({\text {Text}}={\text {Undefined}}) \wedge {\mathbb {I}}({\text {Class}}={\text {regulatory}}{-}{\text {keep-left}})\end{aligned}$$
	$$\begin{aligned}&{\mathbb {I}}({\text {Color}}={\text {Blue}}) \wedge {\mathbb {I}}({\text {Shape}}={\text {Circle}}) \wedge {\mathbb {I}}({\text {Symbol}}={\text {Arrow-Roundabout}}) \\& \wedge {\mathbb {I}}({\text {Text}}={\text {Undefined}}) \wedge {\mathbb {I}}({\text {Class}}={\text {regulatory}}{-}{\text {roundabout}})\end{aligned}$$
	$$\begin{aligned}&{\mathbb {I}}({\text {Color}}={\text {White}}) \wedge {\mathbb {I}}({\text {Shape}}={\text {Circle}}) \wedge {\mathbb {I}}({\text {Symbol}}={\text {Car-Two}}) \\& \wedge {\mathbb {I}}({\text {Text}}={\text {Undefined}}) \wedge {\mathbb {I}}({\text {Class}}={\text {regulatory}}{-}{\text {end-of-no-overtaking}})\end{aligned}$$
	$$\begin{aligned}&{\mathbb {I}}({\text {Color}}={\text {White}}) \wedge {\mathbb {I}}({\text {Shape}}={\text {Circle}}) \wedge {\mathbb {I}}({\text {Symbol}}={\text {Car-Truck}})\\& \wedge {\mathbb {I}}({\text {Text}}={\text {Undefined}}) \\ & \wedge {\mathbb {I}}({\text {Class}}={\text {regulatory}}{-}{\text {end-of-no-overtaking-by-heavy-goods-vehicles}})\end{aligned}$$

## Footnotes

1 We mainly focus on probabilistic circuits over categorical random variables. An extension to the continuous ones is standard.

2 Certain concepts are excluded due to political or ethical concerns.

## Code Availability

The code is available at https://github.com/uiuctml/npc-models.
